# The different effects of four adenosine receptors in liver fibrosis

**DOI:** 10.3389/fphar.2024.1424624

**Published:** 2024-09-03

**Authors:** Lan Yang, Zhao-wei Gao, Xi Wang, Xia-nan Wu, Si-min Li, Ke Dong, Xiao-ming Zhu

**Affiliations:** ^1^ Department of clinical diagnose, Tangdu hospital, Air Force Medical University, Xi’an, Shaanxi, China; ^2^ Department of Obstetrics and Gynecology, Hainan Branch of PLA General Hospital, Sanya, China

**Keywords:** liver fibrosis, adenosine, adenosine receptor, hepatic stellate cells, agonist

## Abstract

**Background:**

The adenosine–adenosine receptor pathway plays important roles in the immune system and inflammation. Four adenosine receptors (i.e., A1R, A2AR, A2BR, and A3R) have been identified. However, the roles of these receptors were different in the disease progress and even play opposite roles in the same disease. This study aims to investigate the roles of A1R/A2AR/A2BR/A3R activation in liver fibrosis.

**Methods:**

Intraperitoneal injection of CCl_4_ into C57BL/6 mice was used to induce liver fibrosis in the models. Adenosine receptor agonists CCPA, CGS21680, BAY 60-6583, and namodenoson were used for A1R/A2AR/A2BR/A3R activation, respectively. Alanine aminotransferase (ALT) and aspartate aminotransferase (AST) levels were used to evaluate the liver function. Hematoxylin and eosin (H&E) staining was used to investigate the pathological damage. Masson staining and Sirius Red staining were performed to evaluate the degree of collagen deposition. CCK8 and scratch assays were used to investigate the proliferation and migration ability of hepatic stellate cells (HSCs).

**Results:**

By using liver fibrosis mouse models, we observed that the A1R and A2AR agonists aggravated liver fibrosis, characterized by increasing ALT and AST levels, more serious liver pathological damage, and collagen deposition. However, the A2BR and A3R agonists alleviated liver fibrosis. Moreover, the A1R and A2AR agonist treatment promotes the proliferation and migration of HSC line LX2, while A2BR and A3R agonist treatment inhibited LX2 proliferation and migration. Consistently, A1R and A2AR agonist treatment elevated the expression of α-SMA and Col1α1 in LX2, whereas A2BR and A3R agonist treatment inhibited the expression of α-SMA and Col1α1 in LX2 cells. Additionally, 5′-N-ethyl-carboxamidoadenosine (NECA), a metabolically stable adenosine analog, alleviated liver fibrosis and inhibited LX2 cell activity, proliferation, and migration.

**Conclusion:**

This study demonstrated the different roles of A1R/A2AR/A2BR/A3R during liver fibrosis development via regulating the HSC activity and proliferation.

## 1 Introduction

Fibrosis is a common pathological repair response to injury and a pathological characteristic of chronic injury or inflammatory disease afflicting various organs, such as the liver (Kisseleva and Brenner, 2021) and lungs (Chanda et al., 2019). Liver fibrosis is characterized by excessive deposition of the extracellular matrix (ECM) in the liver (Parola and Pinzani, 2019; Pei et al., 2023). Progressive liver fibrosis impairs the liver structure and function, eventually resulting in cirrhosis and liver failure. Thus, the pathogenesis of liver fibrosis should be understood to develop a therapeutic strategy.

Hepatic stellate cells (HSCs) are the most important effector cells during the process of hepatic fibrosis. HSC activation and proliferation are the most critical events in the pathological progress of liver fibrosis (Pei et al., 2023). HSCs have two physiological states: quiescent and activated. In a normal liver, HSCs are quiescent with low proliferation and collagen synthesis abilities, whereas in a damaged liver, they are activated and transited into ECM-producing myofibroblast-like cells, characterized by increased α-SMA and Col1α1 proliferation ability and expression and contribute to fibrotic progression (Caligiuri et al., 2021). Thus, suppressing the HSC activity is the potential therapeutic approach for liver fibrosis.

Adenosine is an endogenous purine signaling nucleoside generated from adenosine triphosphate (ATP) breakdown, which is released by various cell types in response to stimuli such as hypoxia, injury, and inflammation (Li et al., 2020). The extracellular conversion of ATP to adenosine is mediated by an enzymatic cascade, including CD39 and CD73, which are expressed on the surface of various cell types in the liver (Tiwari-Heckler and Jiang, 2019). Circulating adenosine and adenosinergic signaling has been implicated in the development and progress of liver injury and hepatic fibrosis (Park et al., 2024). The effects of adenosine are exerted by binding to adenosine receptors (Peleli et al., 2017). There are four adenosine receptors, i.e., A1R, A2AR, A2BR, and A3R. Transcriptional regulation of adenosine receptor expression has been described during experimental liver fibrosis in mouse and human cirrhosis, suggesting their potential involvement in fibrosis development (Fausther, 2018). However, the roles of adenosine receptors during liver diseases are still controversial. For example, the results of the effects of A2AR in liver diseases are conflicting. Chan et al. study reported that A2AR plays a triggering role in the pathogenesis of hepatic fibrosis via increased collagen I expression (Chan et al., 2006). Chiang et al. also demonstrated that A2AR antagonists could prevent and reverse the ability of ethanol to exacerbate liver fibrosis (Chiang et al., 2013). Likewise, A2AR activation promotes collagen production in HSCs and increases HSC proliferation (Che et al., 2007; Ahsan and Mehal, 2014). Conversely, Li et al. revealed that A2AR and A2BR downregulation induced liver and lung fibrotic diseases, whereas their upregulation attenuated fibrotic responses (Li et al., 2024). Imarisio et al. reported that A2AR stimulation could prevent hepatocyte lipotoxicity and non-alcoholic steatohepatitis-associated liver inflammation and fibrosis in rats (Imarisio et al., 2012). These conflicting results indicated that the adenosine receptor function in liver fibrosis is complex. In addition to A2AR, A1R was also involved in activating HSCs and liver fibrosis (Yang et al., 2015; Yang et al., 2010).

Therefore, this study aimed to comprehensively evaluate the functions of four adenosine receptors (A1R, A2AR, A2BR, and A3R) during the process of liver fibrosis and to assess whether and which adenosine receptor could be a remarkable therapeutic target for hepatic fibrosis. Here, we showed the different effects of A1R, A2AR, A2BR, and A3R activation in liver fibrosis.

## 2 Materials and methods

### 2.1 Liver fibrosis mouse models

The intraperitoneal (i.p.) injection of CCl_4_ in C57BL/6 mice was used to induce liver fibrosis, which is a typical mouse model for liver fibrosis study. C57BL/6 mice (male; age: 6–8 weeks; weight: 20–22 g) were purchased from an experimental animal center, Air Force Medical University. The mice were randomly divided into different groups: control group—olive oil (MACKLIN, China, Cat: 8001-25-0), 1 mL/kg i.p.; CCl_4-_induced group [25% CCl_4_ (MACKLIN, China, Cat: 56-23-5) (CCl_4_: olive oil = 1:3), 1 mL/kg i.p.]; NECA treatment group [1 mL/kg i.p. 25% CCl_4_ plus 0.1 mg/kg i.p. NECA (GlpBio, Montclair, CA, United States, Cat: GC15304)]; A1R agonist group [1 mL/kg i.p. 25% CCl_4_ plus 0.5 mg/kg i.p. CCPA (GlpBio, Montclair, CA, United States, Cat: GC45773)]; A2AR agonist group [1 mL/kg i.p. 25% CCl_4_ plus 1 mg/kg i.p. CGS21680 (GlpBio, Montclair, CA, United States, Cat: GC10172)]; A2BR agonist group [1 mL/kg i.p. 25% CCl_4_ plus 4 mg/kg i.p. BAY-606583 (TargetMol, China, Cat: 910487-58-0)]; and A3R agonist group [1 mL/kg i.p. 25% CCl_4_ plus 200 μg/kg i.p. namodenoson (TargetMol, China, Cat: 163042-96-4)]. The chemical structures of CCPA, CGS21680, BAY 60-6583, and namodenoson are given in Supplementary Figure S1. The i.p. injection of CCl_4_ was performed twice weekly for 6 weeks. The other treatment was performed twice weekly from the third week after i.p. CCl_4_. After the last injection, all mice were made to fast overnight. Then, the liver tissues and blood from these mouse models were collected. All experiments were performed according to the animal welfare guidelines implemented by the Institutional Animal Care and Use Committee of the Air Force Medical University.

### 2.2 Liver function test

According to the standard test procedures in the clinical laboratory, the alanine aminotransferase (ALT) and aspartate aminotransferase (AST) levels in serum from the mouse models were detected by using the diagnostic reagent (Shanghai Kehua, China) in an automatic biochemical analyzer (Beckman Coulter, AU5800, Germany).

### 2.3 Pathologic analysis

The liver tissues were fixed in 4% formalin solution and embedded in paraffin; then, the formalin-fixed paraffin-embedded (FFPE) sections were prepared (5 μm thick). The FFPE sections were stained with hematoxylin and eosin (H&E) and Masson and Sirius Red staining according to standard procedures. H&E staining was used to evaluate the necrosis, degeneration, and inflammatory infiltration. Masson staining and Sirius Red staining were used to evaluate the content and degree of collagen fiber in the hepatic portal area. PANNORAMIC and CaseViewer 2.4 software (3DHISTECH, Hungary) were used for image acquisition and analysis.

### 2.4 Quantitative real-time PCR analysis

The total RNA from cells and liver tissues was extracted by using the TRIzol Reagent (Takara, Japan, Cat: 9109). The cDNA was generated by using the PrimeScript™ RT Master Mix (Accubate Biology, China, AG11728). A quantitative real-time PCR (qRT-PCR) system was prepared using the BlasTaq™ 2X qPCR Master Mix (ABM, Canada, Cat: G891). The reaction was performed on a Qiagen Amplifier (Rotor-gene Q MDx 5, Germany). All operations were carried out according to the manufacturer’s instructions. The mRNA expression levels in different groups were calculated by using GAPDH expression as an internal control. The primer sequences are listed in Table 1.

**TABLE 1:** Primer sequence.

Gene	Forward primer (5′-3′)	Reverse primer (5′-3′)
Mouse		
α-SMA	ACC​CAG​CAC​CAT​GAA​GAT​CA	TCT​GCT​GGA​AGG​TAG​ACA​GC
Col1α1	TCC​CTG​GAA​TGA​AGG​GAC​AC	CTC​TCC​CTT​AGG​ACC​AGC​AG
GAPDH	TGG​AAA​GCT​GTG​GCG​TGA​TG	TGG​GGG​TAG​GAA​CAC​GGA​A
Human		
α-SMA	AGC​CAA​GCA​CTG​TCA​GGA​AT	TTG​TCA​CAC​ACC​AAG​GCA​GT
Col1α1	GCC​AAG​ACG​AAG​ACA​TCC​CA	GGC​AGT​TCT​TGG​TCT​CGT​CA
GAPDH	AAA​TCA​AGT​GGG​GCG​ATG​CT	CAA​ATG​AGC​CCC​AGC​CTT​CT

### 2.5 Immunohistochemical analysis

The immunohistochemical (IHC) analysis for liver FFPE sections was performed to detect α-SMA and Col1α1 expressions. In short, the anti-Col1α1 antibody (1:100 dilution; Servicebio, GB11022-3) and anti-α-SMA antibody (1:1,000 dilution; Abcam, ab124964) were used as the primary antibodies. The HRP-conjugated anti-rabbit secondary antibody (1:2,000 dilution; ZSGB-Bio, ZB-2301) was used as the secondary antibody. PANNORAMIC and CaseViewer 2.4 (3DHISTECH, Hungary) were used for image acquisition and analysis. ImageJ was used to calculate the positive areas.

### 2.6 Cell culture

Human hepatic stellate cell line LX2 was cultured in Dulbecco’s modified Eagle medium (DMEM; Gibco, Cat: 12800-017) plus 10% fetal bovine serum (FBS; ExCell Bio, Cat: FSS500) at 37°C with 5% CO_2_ in a humidified incubator.

### 2.7 Cell proliferation assay

The cell counting kit-8 (CCK-8) assay was used to investigate the effects of A1R/A2AR/A2BR/A3R agonists and NECA treatment on LX2 cell proliferation. LX2 cells were uniformly seeded into a 96-well plate, with 2,000 cells in each well. The CCK-8 assay was used to investigate the cell viability at different time points. The CCK-8 reagent was purchased from GlpBio, United States (Cat: GSK10001). Cells were set as control and treatment groups [A1R agonist: CCPA (100 μM); A2AR agonist: CGS21680 (10 μM); A2BR agonist: BAY 60-6583 (10 μM); and A3R agonist: namodenoson (10 nM) and NECA (10 μM)]. At different time points (i.e., 0, 24, 48, and 72 h), 10 μL of the CCK-8 reagent was added into each well, and the cells were incubated for 2 h at 37°C. Then, the OD_450_ value was measured, and the relative cell viability was calculated as a percentage using the formula (mean OD_450_ of cells at different time points/mean OD_450_ of cells at 0 h) × 100 %.

### 2.8 Cell cycle and apoptosis analysis

Flow cytometry (FCM) was used to analyze the cell cycle and apoptosis. For cell-cycle analysis, a cell-cycle detection kit (4A Biotech Co., Ltd., Beijing, China, Cat: FXP0211) was used to treat the cells following the manufacturer’s instructions. In brief, cells were fixed for 2 h with ethanol and stained with propidium iodide (PI). Agilent NovoCyte was used to assess the cell-cycle distribution. The apoptosis detection kit (4A Biotech Co., Ltd., Beijing, China, Cat: FXP022) was used to detect cell apoptosis. Cells were collected, washed twice with phosphate-buffered saline, and stained with Annexin V-FITC and PI in the dark at room temperature for 15 min. Agilent NovoCyte was used to calculate the percentage of apoptotic cells.

### 2.9 Scratch assay

The scratch assay was used to investigate the effects of the adenosine receptor agonist on the LX2 cell migration ability. Control and agonist-treated cells were cultured in a six-well plate. The scratch was performed using a pipette tip. Once the scratch was made, the cells were gently washed with PBS twice, following culture with the serum-free medium. Images were captured immediately and 24 h after the scratch was made. The migration ability of cells was mirrored by the relative migration ratio: (Start distant - End distance)/Start distance.

### 2.10 Statistical analysis

All the quantitative results were presented as the mean ± standard deviation (SD). All data analyses were performed by using GraphPad Prism 8.0. The *t*-test was used to analyze the differences between the two independent samples. *p* < 0.05 was considered to be statistically significant.

## 3 Results

### 3.1 A1R and A2AR agonists aggravated liver fibrosis in CCl_4_-induced mice

**FIGURE 1:**
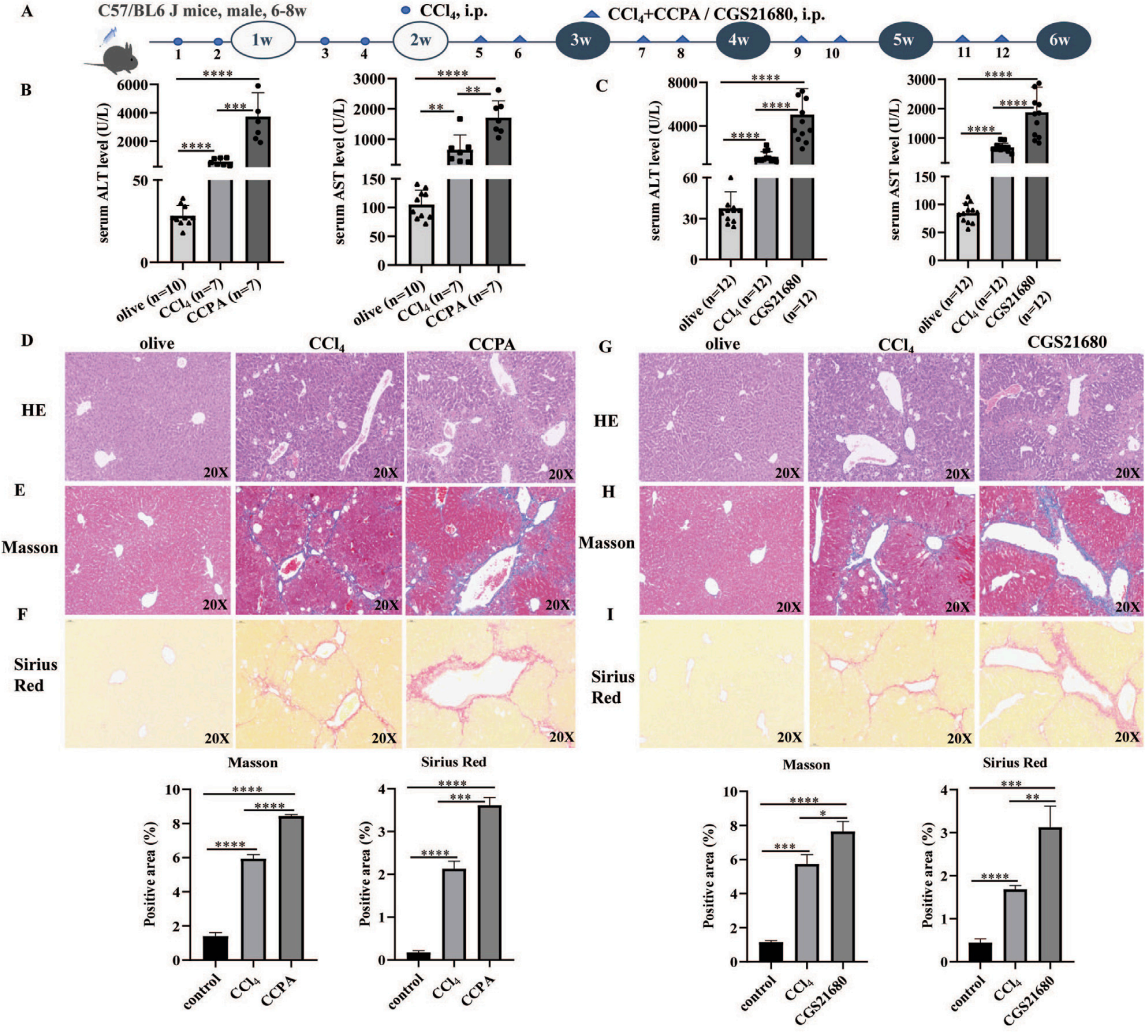
A1R and A2AR agonists aggravated liver fibrosis in CCl_4_-induced mouse models. **(A)** Flowchart of animal experiments. **(B)** Serum ALT and AST levels in control, CCl_4_-induced mice, and CCPA (number of control: CCl_4_: CCAP mice = 10:7:7)-treated mice. **(C)** Serum ALT and AST levels in control, CCl_4_-induced mice, and CGS21680-treated mice. **(D–I)** Representative liver tissue sections of control, CCl_4_-, CCPA-, and CGS21680-treated mice were detected by HE, Masson, and Sirius Red staining. Scale bar: 50 μm; magnification: ×20. The positive area statistics of Masson and Sirius Red staining were measured using ImageJ software. *p < 0.05, **p < 0.01, ***p < 0.0005, and ****p < 0.0001.

CCl_4_-induced mouse models were used to investigate the effects of A1R and A2AR agonists. The flowchart of the mouse experiment is shown in Figure 1A. Compared with control mice, i.p. CCl_4_ increased serum ALT and AST levels. Moreover, the serum ALT and AST levels were higher in A1R-and A2AR-treated mice than those in the control and CCl_4_-induced mice (Figures 1B, C).

H&E staining showed that liver injury and inflammatory cell infiltration were more severe in the A1R-treated CCl_4_ mice (Figure 1D). Masson and Sirius Red staining indicated that the A1R agonist promoted collagen deposition in liver tissues (Figures 1E, F). Similarly, the pathological analysis of liver tissues from mouse models also demonstrated that the A2R agonist aggravated liver injury, immune cell infiltration, and collagen deposition (Figures 1G–I).

### 3.2 A2BR and A3R agonists alleviated liver fibrosis in CCl_4_-induced mice

**FIGURE 2:**
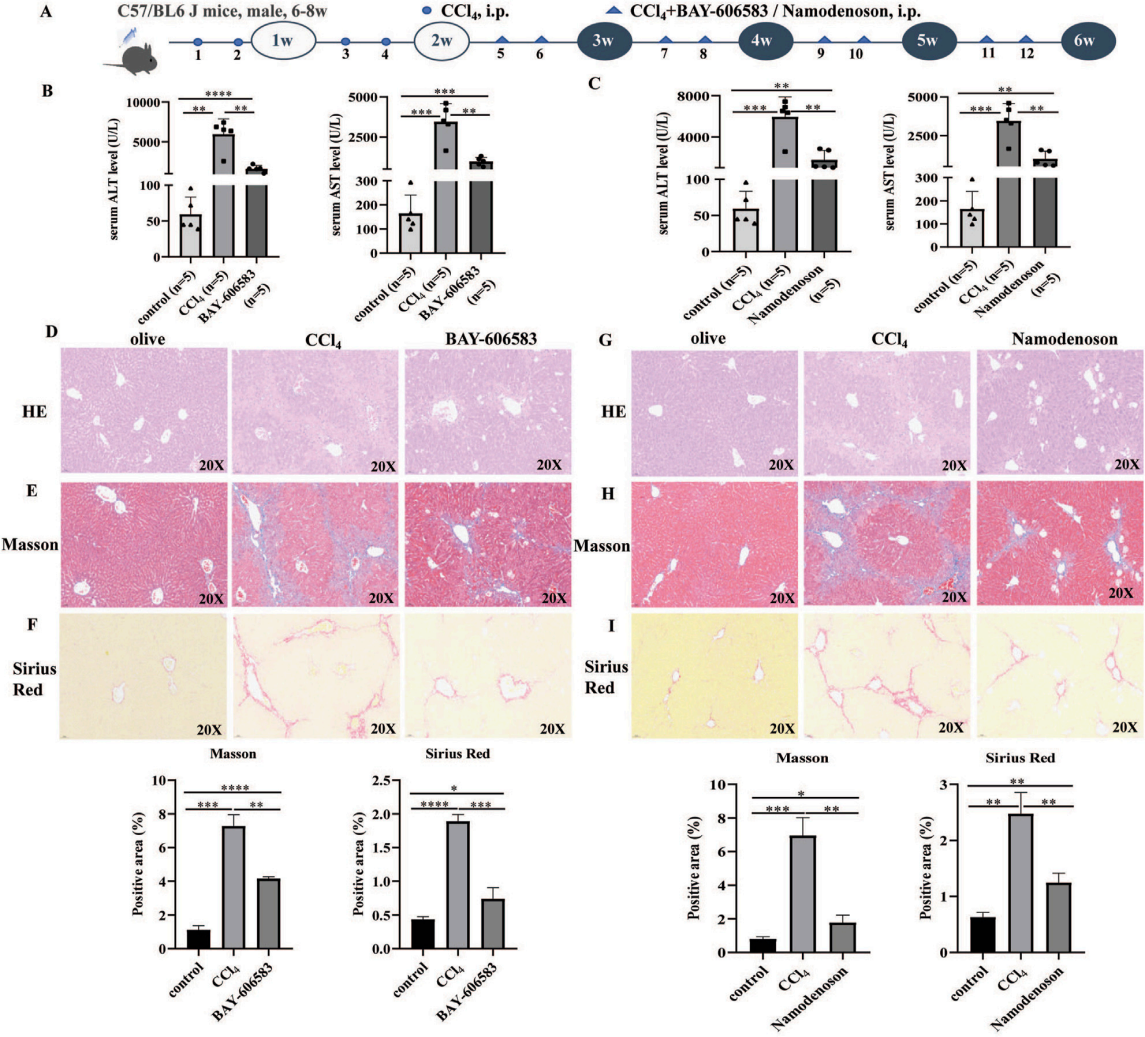
A2BR and A3R agonists alleviated liver fibrosis in CCl_4_-induced mouse models. **(A)** Flowchart of animal experiments. **(B,C)** Serum ALT and AST levels in control, CCl_4_-induced mice, BAY 60-6583-, and namodenoson-treated mice. **(D–I)** Representative liver tissue sections of control, CCl_4_-, BAY 60-6583-, and namodenoson-treated mice were detected by HE, Masson, and Sirius Red staining. Scale bar: 50 μm; magnification: ×20. The positive area statistics of Masson and Sirius Red staining were measured using ImageJ software. *p < 0.05, **p < 0.01, ***p < 0.0005, and ****p < 0.0001.

The effects of A2BR and A3R agonists in liver fibrosis were further investigated. The animal experimental procedure is shown in Figure 2A. Compared with i.p. CCl_4_ mice, the A2BR and A3R agonist treatment significantly reduced the ALT and AST levels (Figures 2B, C). Moreover, the pathological section staining (H&E, Masson, and Sirius Red) of liver tissues revealed that the A2BR agonist reduced liver injury and collagen deposition (Figures 2D–F). Similarly, the A3R agonist also alleviated liver fibrosis disease activity in CCl_4_-induced mouse models (Figures 2G–I), characterized by decreased ALT and AST levels and pathological liver damage.

### 3.3 Effects of the A1R/A2AR/A2BR/A3R agonist on HSC activation, proliferation, and migration

**FIGURE 3:**
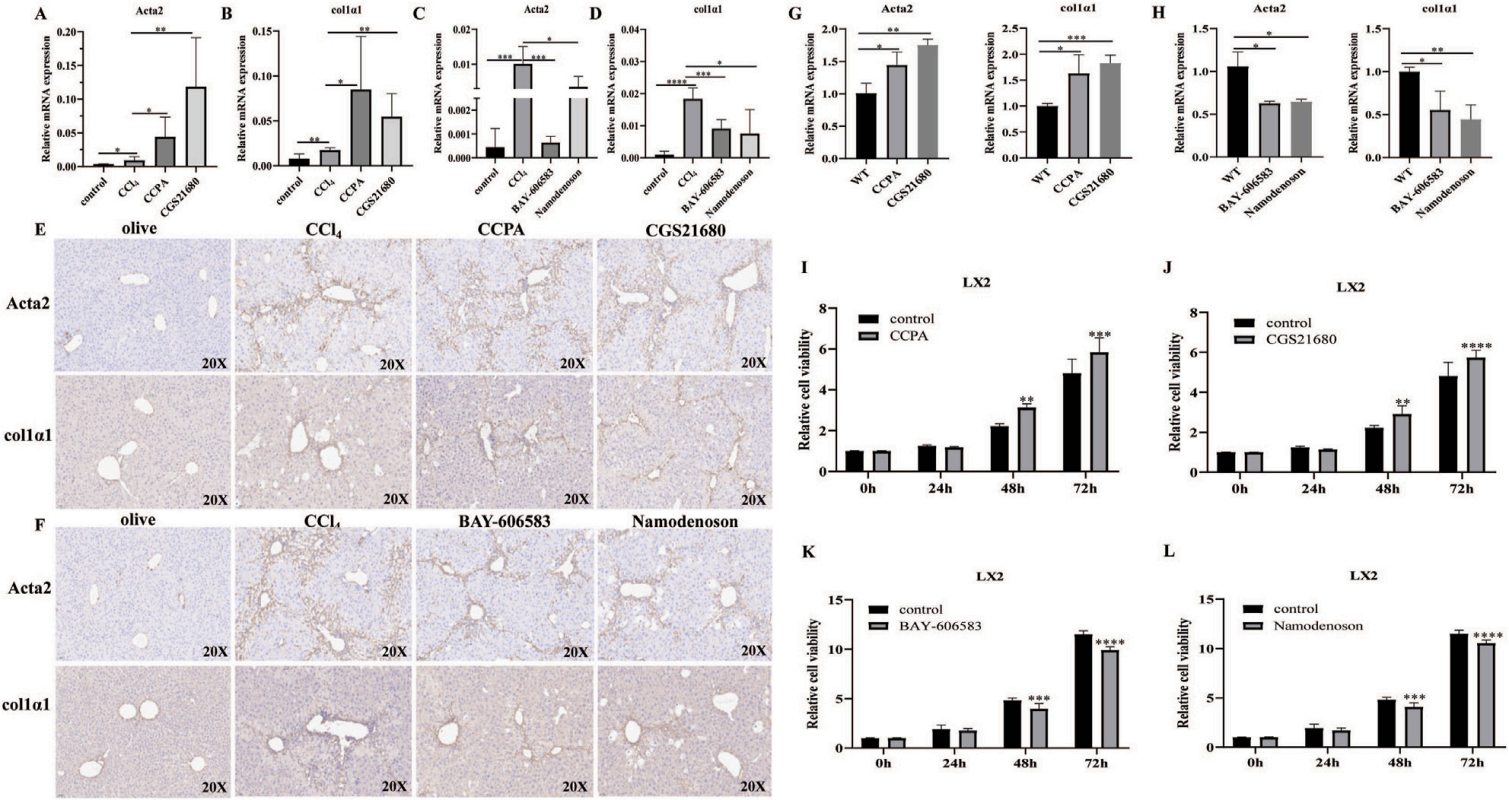
Effects of the A1R/A2AR/A2BR/A3R agonist on HSC activation and proliferation. **(A–F)** qRT-PCR and IHC analysis showed the expression levels of α-SMA (encoded by the *ACTA2* gene) and Col1α1 in liver tissues from control, CCl_4_-_,_ CCPA-, CGS21680-, BAY 60-6583-, and namodenoson-treated mice. Scale bar: 50 μm; magnification: ×20. **(G,H)** qRT-PCR showed the expression levels of α-SMA and Col1α1 in LX2 cells. **(I–L)** CCK-8 assay showed the effects of CCPA, CGS21680, BAY 60-6583, and namodenoson treatment on LX2 cell proliferation. *p < 0.05, **p < 0.01, ***p < 0.0005, and ****p < 0.0001.

To further explore the potential mechanism of adenosine receptors on liver fibrosis, the effects of A1R/A2AR/A2BR/A3R agonist treatment on HSC activity markers in liver tissues were investigated. Compared with CCl_4_ mice, the mRNA expressions of α-SMA and Col1α1 were significantly increased in the A1R/A2AR-treated mice (Figures 3A, B), whereas α-SMA and Col1α1 levels were decreased in the A2BR/A3R-treated mice (Figures 3C, D). Furthermore, IHC results also revealed the promoting effects of A1R/A2AR agonists and inhibiting effects of A2BR/A3R agonists on α-SMA and Col1α1 protein expression (Figures 3E, F).

Moreover, the A1R and A2AR agonists increased the SMA and Col1α1 expressions, whereas the A2BR and A3R agonists inhibited the α-SMA and Col1α1 expressions in LX2 cells (Figures 3G, H). We further investigated the effects of the A1R/A2AR/A2BR/A3R agonist on HSC proliferation *in vitro*. The results revealed that the A1R and A2AR agonists significantly promoted LX2 cell proliferation, whereas the A2BR and A3R agonists inhibited LX2 cell proliferation (Figures 3I–L). Notably, FCM analysis demonstrated that A2BR and A3R agonists promote LX2 cell apoptosis (Supplementary Figure S2A). Moreover, FCM analysis revealed that A1R and A2AR agonist treatment significantly increased the S phase (Supplementary Figure S2B). Collectively, these results indicated that the promoting effects of A1R and A2AR agonists on LX2 proliferation might be related to cell-cycle changes. The inhibiting effects of A2BR and A3R agonists on LX2 proliferation might be associated with increased cell apoptosis. Moreover, the effects of adenosine receptor activation on LX2 cell migration were also investigated. The A1R and A2AR agonists promoted the cell migration ability, whereas the A2BR and A3R agonists inhibited the migration ability of LX2 cells (Supplementary Figure S3).

### 3.4 NECA inhibited HSC activation and alleviated liver fibrosis

**FIGURE 4:**
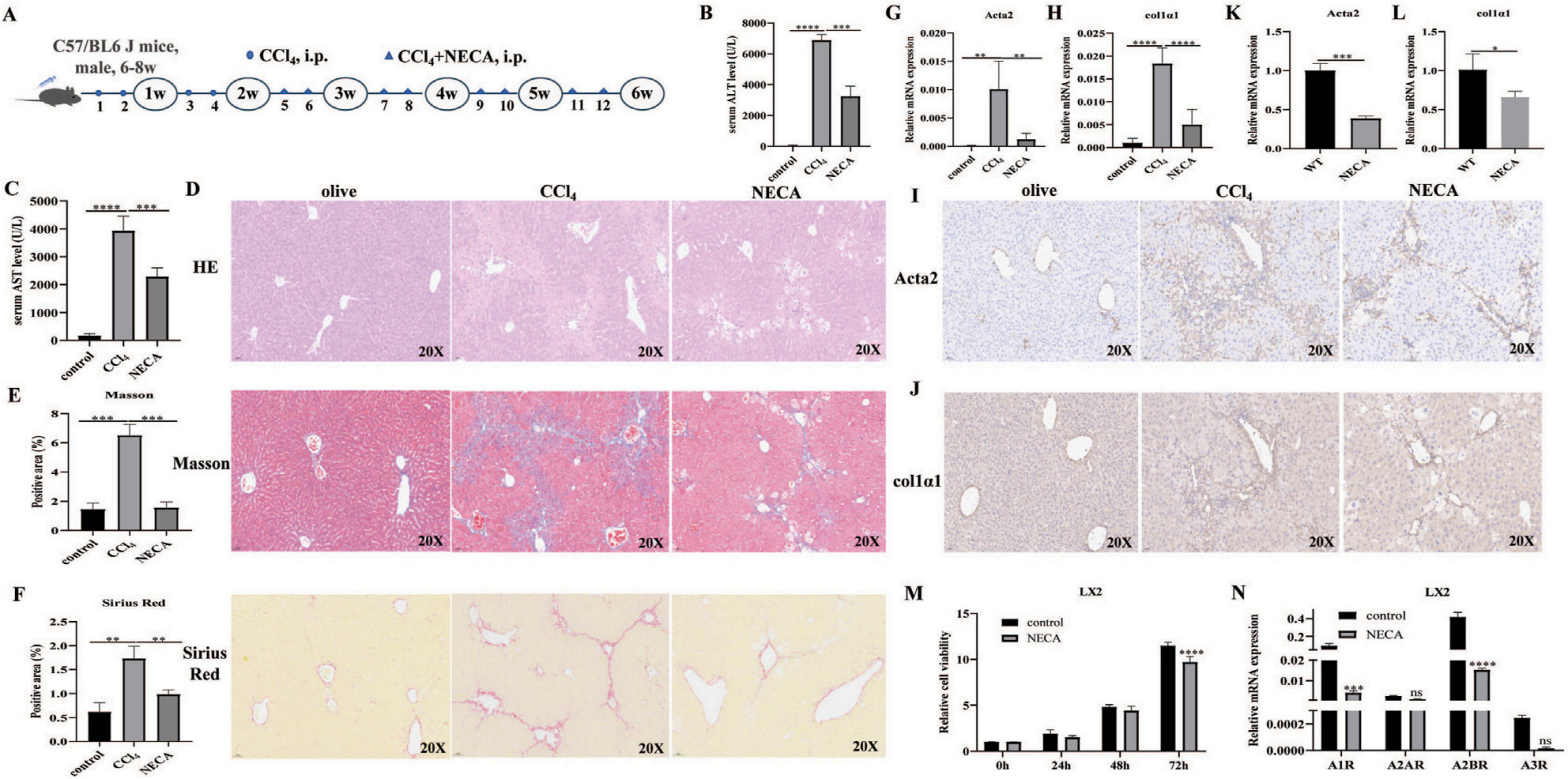
NECA inhibited HSC activation and alleviated liver fibrosis. **(A)** Flowchart of animal experiments. **(B,C)** Serum ALT and AST levels in control, CCl_4_-induced mice, and NECA-treated mice. **(D–F)** Representative liver tissue sections of control, CCl_4_-, and NECA-treated mice were detected by HE, Masson, and Sirius Red staining. Scale bar: 50 μm; magnification: ×20. **(G–J)** qRT-PCR and IHC analysis showed the expression levels of α-SMA and Col1α1 in control, CCl_4_-, and NECA-treated mice. Scale bar: 50 μm; magnification: ×20. The positive area statistics of Masson and Sirius Red staining were measured using ImageJ software. **(K,L)** qRT-PCR analysis showed the expression levels of α-SMA and Col1α1 in LX2 cells. **(M)** Effect of NECA treatment on LX2 cell proliferation. **(N)** Expression of A1R, A2AR, A2BR, and A3R in LX2 cells with or without NECA treatment. *p < 0.05, **p < 0.01, ***p < 0.0005, and ****p < 0.0001; ns: no significance.

As the effects of A1R/A2AR/A2BR/A3R agonist treatment on liver fibrosis differed, the effects of NECA (a metabolically stable adenosine analog) i.p. were further investigated in CCl_4_-induced mouse models (Figure 4A). The results revealed that ALT and AST levels were decreased in NECA-treated mice (vs. CCl_4_-induced mice; Figures 4B,C). H&E staining showed that NECA treatment reduced liver injury (Figure 4D). Masson and Sirius Red staining demonstrated the decreased degree of collagen fibers in the liver tissues from i.p. NECA mice (Figures 4E, F). The α-SMA and Col1α1 expressions were inhibited by the NECA treatment in liver tissues, at mRNA and protein levels (Figures 4G–J). Moreover, the NECA treatment significantly inhibited SMA and Col1α1 expressions in LX2 cells (Figures 4K,L). The effects of NECA on HSC proliferation *in vitro* were further investigated. The results revealed that NECA significantly inhibited LX2 cell proliferation (Figure 4M). Moreover, the scratch assay showed that NECA inhibited LX2 cell migration (Supplementary Figure S3).

### 3.5 Different expression levels of A1R/A2AR/A2BR/A3R in HSCs

As the effects of A1R/A2AR/A2BR/A3R agonists on liver fibrosis severity differed, the expression levels of A1R/A2AR/A2BR/A3R in LX2 cells were investigated. The results revealed that A2BR was the highest-expressed gene among the four adenosine receptors. Notably, the expression levels of A1R/A2AR/A2BR/A3R in LX2 cells were all decreased by the NECA treatment. Moreover, A2BR was still the highest-expressed gene after the NECA treatment (Figure 4N). These results suggest that similar effects between NECA and A2BR, which both alleviate liver fibrosis, might be associated with the highest A2BR expression levels in HSCs.

## 4 Discussion

The adenosine–adenosine receptor pathway plays an important role in the inflammatory response. Currently, four adenosine receptors, i.e., A1R, A2AR, A2BR, and A3R, have been identified. These four adenosine receptors might play different roles in the progression of various diseases and even in the same disease. In this study, A1R and A2AR agonists were found to aggravate liver fibrosis in CCl_4_-induced mouse models, characterized by increased serum ALT and AST levels, increased pathological damage, and collagen deposition in liver tissues. Consistently, LX2 proliferation, migration, and activation were significantly promoted by the A1R and A2AR agonist treatment. However, the A2BR and A3R agonist treatment significantly alleviated liver fibrosis in mouse models and inhibited LX2 proliferation and activation. These results revealed the different roles of various adenosine receptors during liver fibrosis progression. Remarkably, the effects of NECA treatment on liver fibrosis *in vivo* and LX2 cells *in vitro* were similar to those of A2BR and A3R agonists.

Although all are adenosine receptors, accumulated evidence has shown different contributions of A1R/A2R/A2BR/A3R during disease development. First, adenosine receptors were involved in progression of multiple diseases. Studies have demonstrated the function of A1R in brain diseases, including epilepsy (Masino et al., 2011; Amorim et al., 2016), Alzheimer’s disease (Rivera-Oliver and Díaz-Ríos, 2014), Parkinson’s disease (Rivera-Oliver and Díaz-Ríos, 2014; Jakova et al., 2022), and stroke (Liston et al., 2022). A1R has been identified as a promising therapeutic target for non-opioid analgesic agents to treat neuropathic pain (Draper-Joyce et al., 2021). A2AR, A2BR, and A3R were mostly involved in cancer development (Shi et al., 2019; Willingham et al., 2018; Cekic et al., 2012; Vecchio et al., 2016; Harvey et al., 2020), inflammation (Mediero et al., 2015; Barletta et al., 2012; D'Antongiovanni et al., 2020), and cardiac disease (Lu et al., 2008), among others. Second, the roles of these adenosine receptors were different even in the same disease. For example, in hepatic ischemia/reperfusion (IR) injury, the A2AR agonist protected the primary steatotic murine hepatocytes from IR damage. By contrast, the A1R agonist enhanced IR damage, intracellular steatosis, and oxidative species production (Alchera et al., 2021). Studies have reported the opposite function between A1R and A2AR in central and peripheral nervous systems (Deb et al., 2019; Melani et al., 2006).

Zhu et al. reported that in hepatic disease, liver-specific depletion of A1R aggravated, whereas overexpression attenuated diet-induced metabolic-associated steatohepatitis in mice by regulating sterol regulatory element-binding protein maturation (Zhu et al., 2024). However, Arroyave-Ospina JC reported that A1R antagonism could protect against lipotoxicity in metabolic dysfunction-associated liver disease; similarly, the A1R agonist abolished the protective effects of caffeine (Arroyave-Ospina et al., 2023). Notably, Yang et al. (2010) reported that mice lacking A1R were protected from developing liver fibrosis induced by CCl_4_, which is consistent with the aggravated effects of the A1R agonist in CCl_4_-induced liver fibrosis in this study. For A2AR, tumor-promoting and -inhibiting effects in hepatocellular carcinoma (HCC) have been reported. Allard B et al. demonstrated that A2AR is a tumor suppressor of non-alcoholic steatohepatitis (NASH)-associated HCC. A2AR knockout could promote the development of spontaneous and carcinogen-induced HCC in mice (Allard et al., 2023). However, Ma XL et al. reported the suppressive effects of A2AR blockage on HCC growth and metastasis (Ma et al., 2019). Additionally, Myojin et al. found that A2AR inhibition could increase the anti-tumor efficacy of anti-PD1 treatment in HCC mouse models, which also indicated the anti-cancer effects of A2AR blockage (Myojin et al., 2024). In NASH, accumulated evidence reported that A2AR stimulation could prevent NASH development in mouse models via the multilevel inhibition of signals that cause lipotoxicity and inflammation (Imarisio et al., 2012; Zhou et al., 2019; Alchera et al., 2017). However, Chiang DJ revealed that in alcoholic liver fibrosis, the A2AR antagonist prevented and reversed liver fibrosis in ethanol-exacerbated liver fibrosis mouse models by suppressing HSC activation (Chiang et al., 2013). In addition to A2AR, studies have reported that A2BR or A3R stimulation could ameliorate the fibrotic progress in NASH mouse models (Li et al., 2023; Fishman et al., 2019). Collectively, these studies demonstrated that the roles of adenosine receptors were complex and multifaceted in liver disease models with different backgrounds.

## 5 Conclusion

In conclusion, our study demonstrated the different effects of A1R, A2AR, A2BR, and A3R in liver fibrosis, which might regulate the HSC activation at least in part. Due to the complex roles of adenosine receptors in liver fibrosis, the safety and potential side effects of adenosine receptor target therapy need more attention in the clinical trial study.

## Data Availability

The raw data supporting the conclusion of this article will be made available by the authors, without undue reservation.

## Supplementary material

SUPPLEMENTARY FIGURE S1 The chemical structures of CCPA, CGS21680, BAY 60-6583, and namodenoson.

SUPPLEMENTARY FIGURE S2 FCM analysis showed the effects of the A1R/A2AR/A2BR/A3R agonist and NECA on cell apoptosis and cycles of LX2 cells. (A) Cell apoptosis detection. (B) Cell cycle detection. **p* < 0.05, ***p* < 0.01, ****p* < 0.0005, and *****p* < 0.0001.

SUPPLEMENTARY FIGURE S3 Scratch assay showed the effects of the A1R/A2AR/A2BR/A3R agonist and NECA on the migration ability of LX2 cells. Scale bar: 1,000 μm; magnification: ×4. The migration length was measured using ImageJ software. **p* < 0.05, ***p* < 0.01, ****p* < 0.0005, and *****p* < 0.0001.
