# Supplementary material for: The different effects of four adenosine receptors in liver fibrosis
Source: Front Pharmacol. 2024 Sep 3;15:1424624. doi: 10.3389/fphar.2024.1424624 (PMC11405188; doi:10.3389/fphar.2024.1424624)
Supplement: Supplementary file 3 [file DataSheet1.ZIP › files-1/V3-YL The different roles of adenosine receptors in liver fibrosis.docx]

**The different roles of adenosine receptors in liver fibrosis**

**Abstract**

**Keywords**

**Introduction**

Fibrosis is a normal healing response to injury and it is a pathological feature of chronic injury or inflammatory disease afflicting various organ, including liver and lungs. Liver fibrosis is characterized by excessive deposition of extracellular matrix (ECM) in liver, resulting in changes in liver structure and function, Over time, liver fibrosis can progress to cirrhosis, an irreversible liver disease that can lead to liver failure and death. Thus, understanding the pathogenesis of liver fibrosis is essential developing treatment strategies.

Hepatic stellate cells (HSCs) are the most important effector cells in the process of hepatic fibrosis, and HSCs activation and proliferation are the key steps of hepatic fibrosis. Upon liver injury or inflammatory, HSCs convert to ECM producing myofibroblast-like cells, which characterized by increased expression of α-SMA and col1α1, and contribute to liver fibrosis progression. Thus, inactivation of HSCs are key to liver fibrosis regression.

Adenosine is an endogenous nucleoside with increased concentration in the site of tissue injury and hypoxia. Adenosine is involved in a variety of physiological and pathological processes by binding to adenosine receptors. There are four adenosine receptors, i.e. A1R, A2AR, A2BR and A3R. Up liver injury or inflammatory condition, adenosine receptor expression and function are affected, which regulated the activation of HSCs and involved in liver fibrosis progress. However, up to date, the function of adenosine receptors during liver fibrosis are still controversial. This study will comprehensively evaluate the role of four adenosine receptors in the process of liver fibrosis.

1. **Materials and methods**
   1. **Liver fibrosis mice models**

CCl_4_ intraperitoneal injection (i.p.) to C57BL/6 mice were used to induce liver fibrosis, which is a typical models for liver fibrosis study. C57BL/6 mice (male, age: 6-8 weeks, weight: 20-22g ) were purchased from experimental animal center, Air Force Medical University. The mice were randomly divided into different groups (n=8/group): control group: olive oil (公司，货号), 1ml/kg i.p.; model group [25% CCl_4(公司，货号)_ (CCl_4_ : olive oil = 1:3), 1ml/kg i.p.]; NECA treatment group [25% CCl_4_ , 1ml/kg i.p. + NECA (公司货号), xx剂量mg/kg i.p.]; A1R agonist group [25% CCl_4_ , 1ml/kg i.p. + CCPA (公司货号), 4mg/kg i.p.]; A2AR agonist group [25% CCl_4_ , 1ml/kg i.p. + CGS21680 (公司货号), xx剂量mg/kg i.p.]; A2BR agonist group [25% CCl_4_ , 1ml/kg i.p. + BAY-606583 (公司货号), xx剂量mg/kg i.p.]; A3R agonist group [25% CCl_4_ , 1ml/kg i.p. + Namodenoson (公司货号), xx剂量mg/kg i.p.]. The CCl_4_ i.p. was performed twice weekly for 6 weeks. The others i.p. was performed twice weekly from the third week.

After the last injection, all mice fasted overnight. The liver tissues and blood of the mice were collected. All experiments were conducted according to animal welfare guidelines by Air Force Medical University’s Institutional Animal Care and Use Committee.

- 1. **Liver function test**

The alanine aminotransferase (ALT) and aspartate aminotransferase (AST) in serum from mice models were detected by an automatic biochemical analyzer (Beckman Coulter, AU5800, Germany).

- 1. **Pathologic analysis**

Pathological section: Formalin-fixed paraffin-embedded (FFPE) sectionsof liver tissues were prepared (5μm thick). Then hematoxylin and eosin (H&E) staining was performed to evaluate the xxx. Masson staining were performed to investigate the xxx. Sirius red staining were performed to evaluate the xxx. Pannoramic and CaseViewer 2.4 software (3DHISTECH, Hungary) were used for image acquisition and analysis.

- 1. **Quantitative Real-time PCR Analysis (qRT-PCR)**

Total RNA from cells and liver histiocytic suspension were extracted by using Trizol reagent (GLPBIO, Montclair, CA, United States, Cat: xxx). The cDNA was generated by using Prime Script™ RT Master Mix (XXX, Cat: xxx). qRT-PCR reaction system was prepared using BlasTaq qPCR Master Mix (abm, xxx, Cat: xxx). The reaction was performed on Qiagen Amplifer (xxx, xxx). All operation was according to the manufacturer’s instructions. The mRNA expression levels in different groups were calculated using GAPDH expression as an internal control. The primers are listed in Table 1.

- 1. **Immunohistochemical (IHC) analysis**

IHC analysis for liver FFPE tissues was carried out to detect the expression of α-SMA and collagen I. In short, anti-collagen I antibody (1:100 dilution; Servicebio, GB11022-3), and anti- α-SMA antibody (1:1000 dilution; Abcam, ab124964) were used as the primary antibodies. XXX (1:2000 dilution; xxx, Cat: xxx) was used as second antibody. Pannoramic and CaseViewer 2.4 software (3DHISTECH, Hungary) were used for image acquisition and analysis. Image J software was used to calculate the positive areas.

- 1. **Cell Culture**

Human hepatic stellate cell line LX2 was cultured in Dulbecco’s modified Eagle’s medium (DMEM, Gibco, Cat: 12800-017) plus 10% fetal bovine serum (FBS, ExCell Bio, Cat: FSS500) at 37℃ with 5% CO_2_ in a humidified incubator.

- 1. **Cell proliferation assay**

Cell count Kit-8 (CCK8) assay was used to investigate the effects of A1R/A2AR/A2BR/A3R agonist and NECA treatment on LX2 cells proliferation. Cells were uniformly seeded into 96-well plate, with 2000 cells in each well. CCK8 assay was used to investigate the cell viability at different time point. CCK8 reagent was purchased from GLPBIO (Cat: GSK10001,GLPBIO, Montclair, CA, USA). Cells were set into control group and adenosine receptors agonist treatment group (A1R agonist: xxx; A2AR agonist: xxx; A2BR agonist: xxx; A3R agonist: xxx; NECA: xxx). The drug concentration were XXX, XXX, XXX, XXX and xxx respectively.

- 1. **Statistical Analysis**

All quantitative results have been presented as mean ± standard deviation (SD). All data analyses were performed by using GraphPad prism 8.0. T-test was used to analyze the differences between two independent samples. P<0.05 was considered to be statistically significant.

1. **Results**
   1. **A1R and A2AR** **agonist promote aggravated liver fibrosis in CCl4-induced mice**

CCl_4_-induced mice models were used to investigate the effect of A1R and A2AR agonist. The flow chart of mice experiment was shown in Figure 1A. Compared with control mice, CCl_4_ i.p. induced the increased levels of serum ALT and AST. Moreover, the serum ALT and AST were more increased in CCl_4_-induced mice treated by A1R agonist and A2AR agonist (Figure 1B, C).

H&E staining showed that more liver injury and infiltrated inflammatory cells in A1R agonist treated CCl_4_-mice (Figure 1D). Masson and Sirius-red staining indicated that A1R agonist promote the collagen deposition in liver tissues (Figure 1E, F). Similarly, the pathological section results of liver tissues from mice models also showed the A2R agonist aggravated liver injury, immune cell infiltration and collagen deposition (Figure 1G-I)


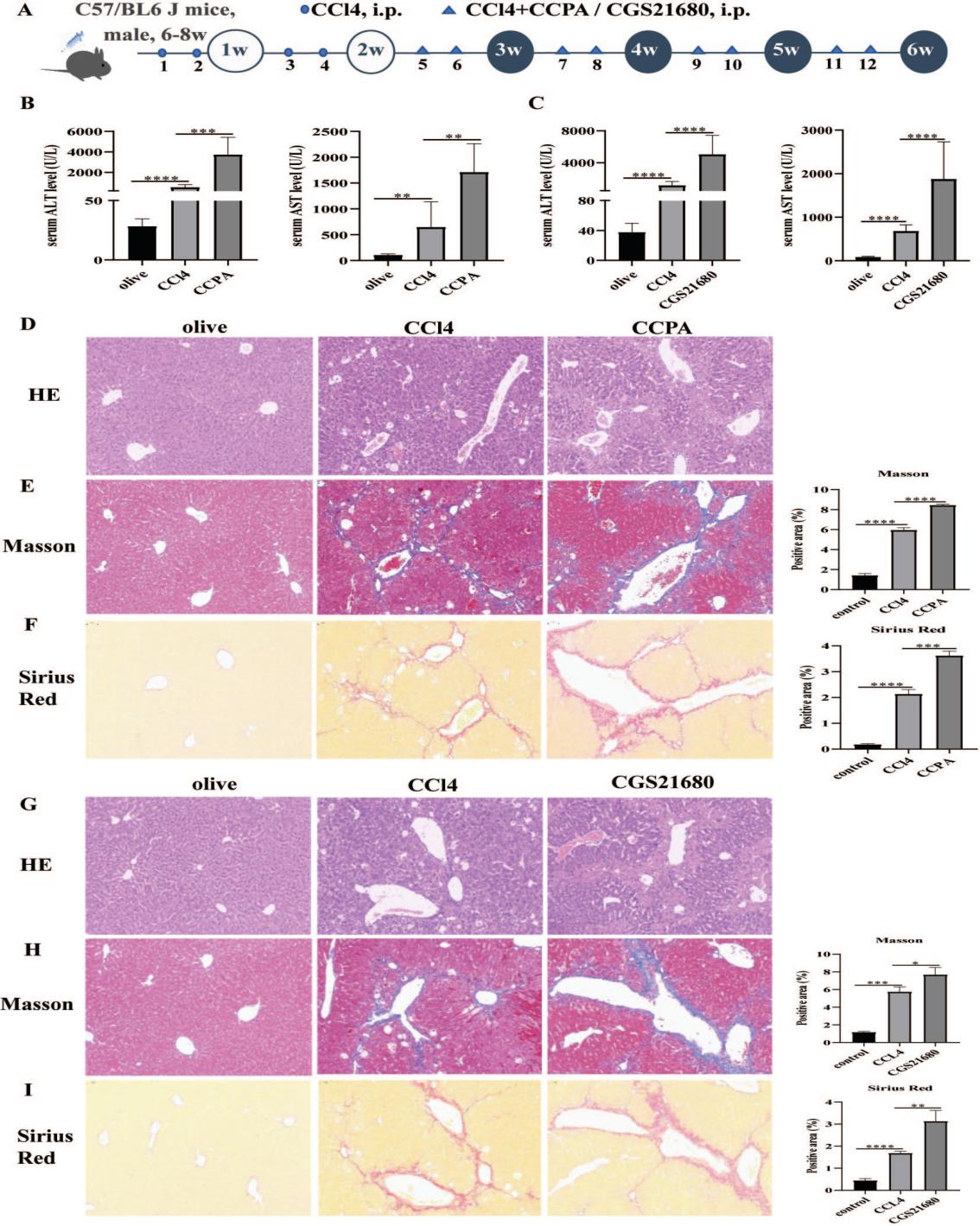


Note: A1R and A2AR agonist promote aggravated liver fibrosis in CCl4-induced mice. A: low chart of animal experiments. B&C: Serum ALT and AST levels in control, CCl_4_-mice, CCPA and CGS21680 treated mice. D&E&F&G&H&I: Representative liver tissue sections of control，CCl_4_，CCPA and CGS21680 treatment group were detected by HE、Masson and Sirius Red staining, scale bar, 50um; magnification, 20x. The positive areas statistic of Masson and Sirius Red staining were measured by Image J software. **P* < 0.05, ***P* < 0.01, ****P* < 0.0005, *****P* < 0.0001.

- 1. **A2BR and A3R agonist alleviated liver fibrosis in CCl4-induced mice**

We further investigated the effect of A2BR and A3R agonist in liver fibrosis. The procedure of animal experiments was shown in Figure 2A. Compared with CCl_4_ i.p. mice, A2BR and A3R agonist treatment significantly reduced the ALT and AST levels in CCl_4_-induced mice (Figure 2B, C). Moreover, The pathological section staining (H&E, masson, sirius-red) of liver tissues showed that A2BR agonist reduced the liver injury and collagen deposition (Figure 2D-F). Similarly, A3R agonist i.p. also alleviated liver fibrosis disease activity in CCl_4_-induced mice models (Figure 2G-I).


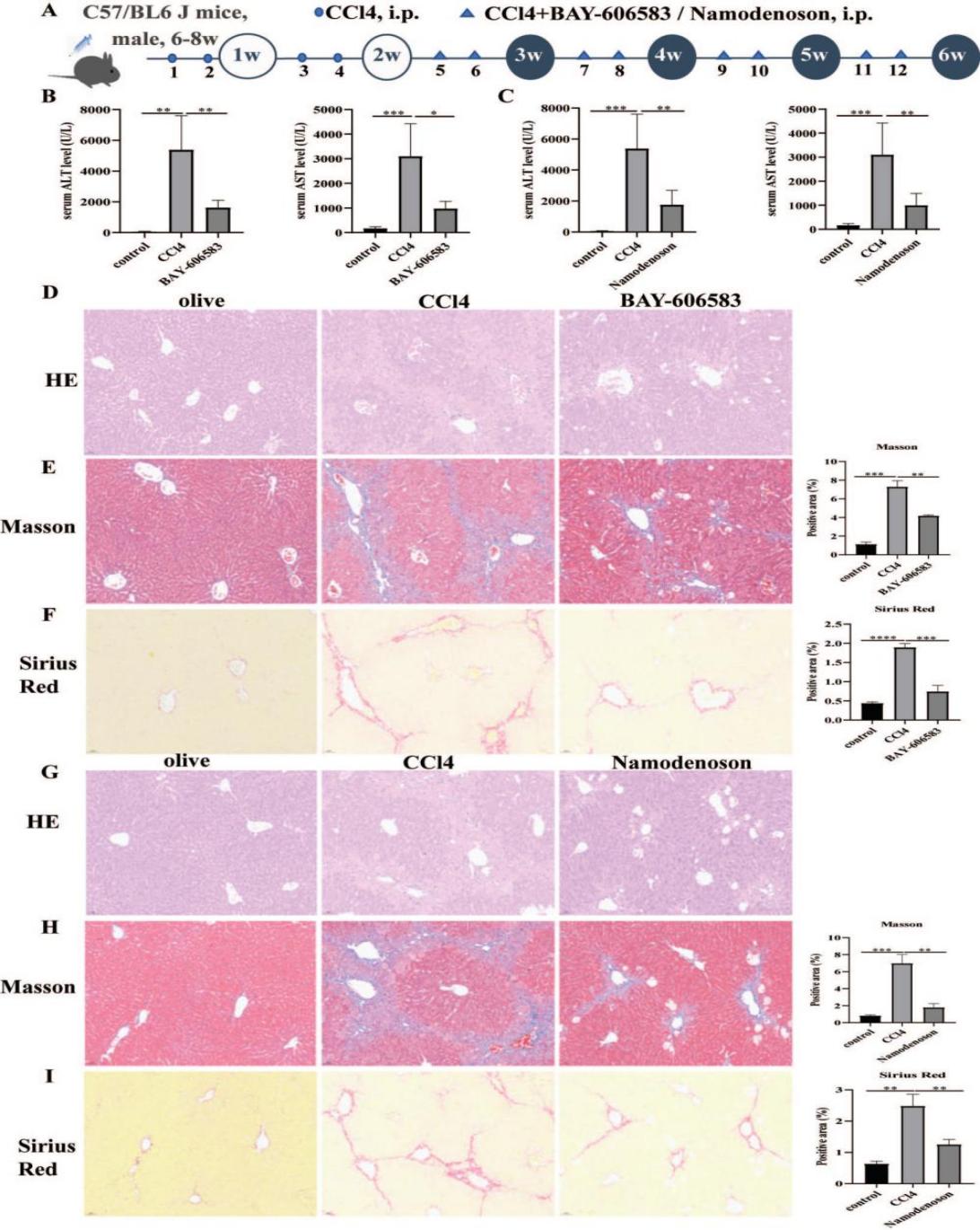


Note: A2BR and A3R agonist alleviated liver fibrosis in CCl4-induced mice. A: low chart of animal experiments. B&C: Serum ALT and AST levels in control, CCl_4_-mice , BAY 60-6583 and Namodenoson treated mice. D&E&F&G&H&I: Representative liver tissue sections of control、CCl_4 ,_BAY 60-6583 and Namodenoson treatment group were detected by HE、Masson and Sirius Red staining, scale bar, 50um; magnification, 20x. The positive areas statistic of Masson and Sirius Red staining were measured by Image J software. **P* < 0.05, ***P* < 0.01, ****P* < 0.0005, *****P* < 0.0001.

- 1. **The effect of A1R/A2AR/A2BR/A3R agonist on HSCs activation and proliferation**

To further explore the potential mechanism of adenosine receptors on liver fibrosis, we investigated the effect of A1R/A2AR/A2BR/A3R agonist i.p. on HSC activation markers in liver tissues. Compared with CCl_4_-mice, the expression of SMA and Col1α-1 were significant increased in A1R/A2AR agonist i.p. mice (Figure 3A, B，E), while SMA and Col1α-1 were decreased in A2BR/A3R agonist i.p. mice (Figure 3C, D, F).

Moreover, the A1R and A2AR agonist elevated the expression of SMA and Col1α-1 in HSCs cell line -LX2, while A2BR and A3R agonist inhibited the expression of SMA and Col1α-1 in LX2 cells (Figure 3G,H).We further investigated the effects of A1R/A2AR/A2BR/A3R agonist on HSCs proliferation in *vitro*. The results showed that the A1R and A2AR agonist significantly promoted the proliferation of LX2 cells, while the A2BR and A3R agonist inhibited the proliferation of LX2 cells (Figure 3I-L).


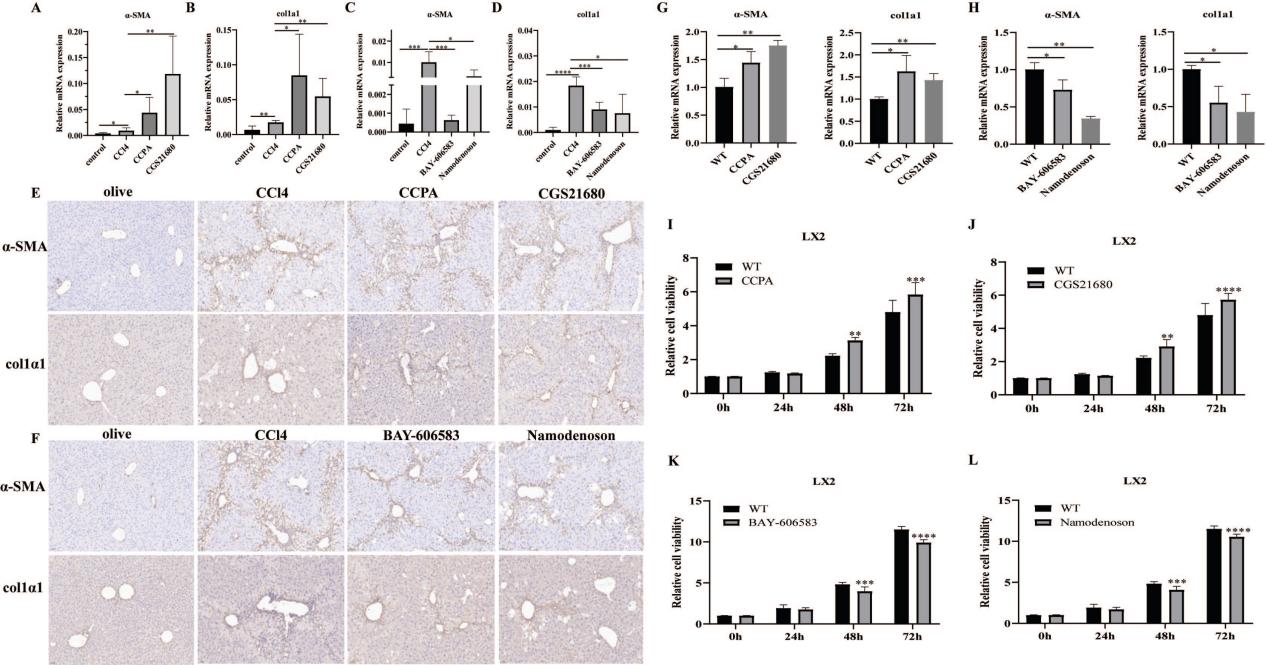


**Note: The effect of A1R/A2AR/A2BR/A3R agonist on HSCs activation and proliferation.** A&B&C&D&E&F**:** The expression levels of α-SMA and col1α1 in olive、CCl_4_ , CCPA, CGS21680, BAY60-6583 and Namodenoson treatment mice were analyzed by qRT-PCR and immunohistochemical (IHC) staining analysis. scale bar, 50μm; magnification, 20x. G&H: The expression levels of α-SMA and col1α1 in LX2 were analyzed by qRT-PCR. I&J&K&L:CCK8 showed the effect of CCPA, CGS21680, BAY60-6583 and Namodenoson treatment on LX2 proliferation. **P* < 0.05, ***P* < 0.01, ****P* < 0.0005, *****P* < 0.0001.

- 1. **NECA inhibited HSCs activation and alleviate liver fibrosis**

As the different effect of A1R/A2AR/A2BR/A3R agonist i.p. on liver fibrosis, we further investigated the function of NECA (a adenosine analogues) i.p. in CCl_4_-induced mice (Figure 4A). The results showed that ALT and AST levels were decreased in NECA i.p. mice (vs CCl_4_-induced mice; Figure 4B, C). H&E staining showed that NECA treatment reduced the liver injury (Figure 4D). Masson and sirius-red staining showed the decreased degree of collagen fiber in the liver from NECA i.p. mice (Figure 4E, F). The expression of SMA and Col1α-1 were inhibited by NECA treatment in liver tissues(Figure 4G-J). Moreover, NECA treatment significantly inhibited the expression of SMA and Col1α-1 in LX2 cells (Figure 4K,L)

We further investigated the effects of NECA on HSCs proliferation in *vitro*. The results showed that the NECA significantly inhibited the proliferation of LX2 cells (Figure 4M).


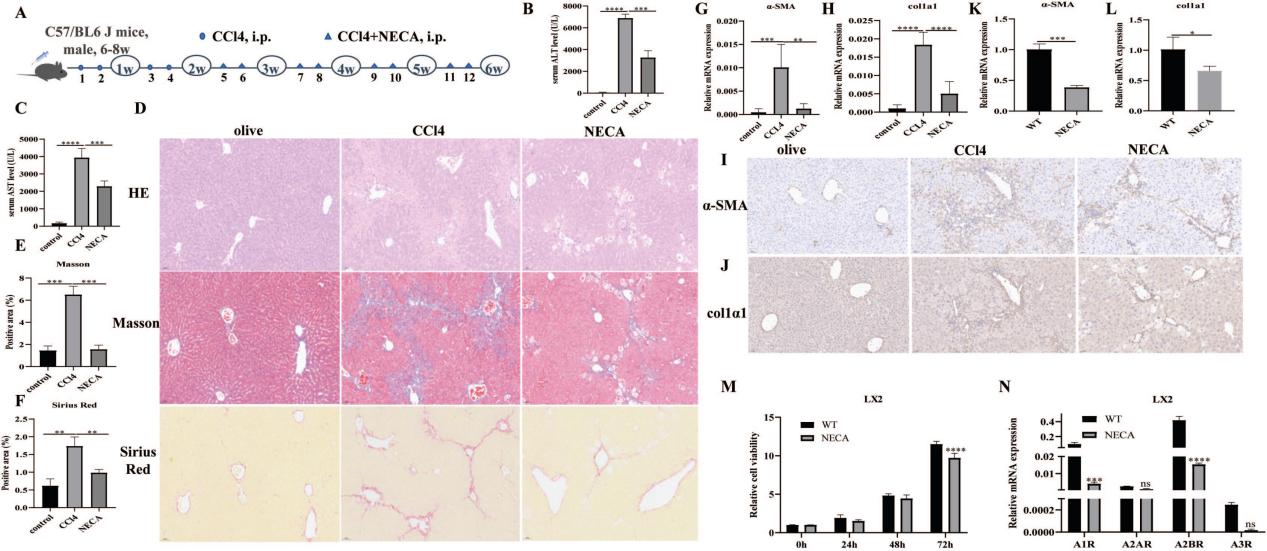


Note: NECA inhibited HSCs activation and alleviate liver fibrosis. A: low chart of animal experiments. B-C: Serum ALT and AST levels in control, CCl_4_-mice and NECA treated mice. D&E&F:: Representative liver tissue sections of control、CCl_4_ and NECA treatment group were detected by HE、Masson and Sirius Red staining, scale bar, 50um; magnification, 20x. G&H&I&J: The expression levels of α-SMA and col1α1 in olive、CCl_4_ NECA treatment mice were analyzed by qRT-PCR and immunohistochemical (IHC) staining analysis.scale bar, 50μm; magnification, 20x. The positive areas statistic of Masson and Sirius Red staining were measured by Image J software. K-L:The expression levels of α-SMA and col1α1 in LX2 were analyzed by qRT-PCR. M:CCK8 showed the effect of NECA treatment on LX2 proliferation. N:The different expression of A1R, A2AR, A2BR and A3R with NECA treatment in LX2 cells were analyzed by qRT-PCR. **P* < 0.05, ***P* < 0.01, ****P* < 0.0005, *****P* < 0.0001.

- 1. **The different expression levels of A1R/A2AR/A2BR/A3R in HSCs**

As the different effect of A1R/A2AR/A2BR/A3R agonist i.p. on the disease severity, we investigated the expression levels of A1R/A2AR/A2BR/A3R in LX2. The results showed that A2BR was the highest expressed gene among the four adenosine receptors. Notably, the expression levels of A1R/A2AR/A2BR/A3R in LX2 were all decreased by NECA treatment. And the A2BR was still the highest expressed genes after NECA treatment (Figure 4N). These results suggests that the similar effects between NECA and A2BR, which both alleviate liver fibrosis, might be associated with the highest expression levels of A2BR in HSCs.
