# Supplementary material for: The different effects of four adenosine receptors in liver fibrosis
Source: Front Pharmacol. 2024 Sep 3;15:1424624. doi: 10.3389/fphar.2024.1424624 (PMC11405188; doi:10.3389/fphar.2024.1424624)
Supplement: Supplementary file 3 [file DataSheet1.ZIP › files-1/V6-YL manuscript The different roles of adenosine receptors in liver fibrosis.docx]

**The different effect of four adenosine receptors in liver fibrosis**

Lan Yang^1^^†^, Zhao-wei Gao^1†^, Xi Wang^1^, Xia-nan Wu^1^, Si-min Li^1^, Ke Dong^1^*, Xiao-ming Zhu^2^*

1 Department of clinical diagnose, Tangdu hospital, Air Force Medical University, Xi’an, Shaanxi province, 710038, China.

2 Department of Obstetrics and Gynecology, Hainan Branch of PLA General Hospital, Sanya, 572022, China.

# These authors contribute equally to this work

* Corresponding author:

Ke Dong, [tdjyk3@fmmu.edu.cn;](mailto:tdjyk3@fmmu.edu.cn;) Xiao-ming Zhu, xiaomingzhu1981@hotmail.com

**Abstract**

**Background:** Adenosine-adenosine receptor pathway play important roles in immune and inflammation. Four adenosine receptors (i.e. A1R, A2AR, A2BR andA3R) have been identified. However, there were different function for these receptors during disease progress, even play opposite roles in the same disease. This study aims to investigate the influence of these four adenosine receptors in liver fibrosis.

**Methods:** Intraperitoneally injected with CCl4 to C57BL/6 mice was used to induce liver fibrosis models. Adenosine receptor agonist CCPA, CGS21680, BAY 60-6583 and Namodenoson were used for A1R/A2AR/A2BR/A3R activation respectively. Alanine aminotransferase (ALT) and aspartate aminotransferase (AST) levels were used to evaluate the liver function. Hematoxylin and eosin (H&E) staining was used to investigate the pathological damage. Masson and Sirius red staining were performed to evaluate the degree of collagen deposition.

**Results:** By using liver fibrosis mice models, our study showed that A1R and A2AR agonist aggravated liver fibrosis, characterized by increasing liver function markers levels, more serious liver pathological damage and collagen deposition. However, A2BR and A3R agonist alleviated liver fibrosis. Moreover, the A1R and A2AR agonist treatment promote the proliferation of hepatic stellate cells (HSCs) cell-LX2, while A2BR and A3R agonist treatment inhibited LX2 proliferation. Consistently, A1R and A2AR agonist treatment elevated the expression of α-SMA and col1α-1 in LX2, while A2BR and A3R agonist treatment inhibited the expression of α-SMA and col1α-1 in LX2 cells. In addition, 5’-N-ethyl-carboxamidoadenosine (NECA), the adenosine analogues, showed the alleviated effect on liver fibrosis and inhibiting effect on LX2 cell’s activity.

**Conclusion:** This study demonstrated the different roles of A1R/A2AR/A2BR/A3R during liver fibrosis development via regulating the HSCs activity.

**Keywords：**liver fibrosis; adenosine; adenosine receptor; hepatic stellate cells; agonist

**Introduction**

Fibrosis is a normal healing response to injury and it is a pathological feature of chronic injury or inflammatory disease afflicting various organ, including liver^[1]^ and lungs^[2]^. Liver fibrosis is characterized by excessive deposition of extracellular matrix (ECM) in liver, resulting in changes in liver structure and function, over time, liver fibrosis can progress to cirrhosis, an irreversible liver disease that can lead to liver failure and death^[3, 4]^. Thus, understanding the pathogenesis of liver fibrosis is essential developing treatment strategies. Hepatic stellate cells (HSCs) are the most important effector cells in the process of hepatic fibrosis, and HSCs activation and proliferation are the key steps of hepatic fibrosis^[4]^. Upon liver injury or inflammatory, HSCs convert to ECM producing myofibroblast-like cells, which characterized by increased expression of α-SMA and col1α1, and contribute to liver fibrosis progression^[5]^ . Thus, suppression the activity of HSCs is the key of target therapy of liver fibrosis.

Adenosine is an endogenous nucleoside with increased concentration in the site of tissue injury and hypoxia^[6]^. Adenosine is involved in a variety of physiological and pathological processes by binding to adenosine receptors^[7]^. There are four adenosine receptors, i.e. A1R, A2AR, A2BR and A3R. Up liver injury or inflammatory condition, adenosine receptor expression and function are affected, which could regulate the activation of HSCs and be involved in liver fibrosis progress^[8]^. However, up to date, the function of different adenosine receptors during human disease are still controversial and even contradictory. This study aims to comprehensively evaluate the role of four adenosine receptors in the process of liver fibrosis.

**1. Materials and methods**

- 1. **Liver fibrosis mice models**

CCl4 intraperitoneal injection (i.p.) to C57BL/6 mice were used to induce liver fibrosis, which is a typical model for liver fibrosis study. C57BL/6 mice (male, age: 6-8 weeks, weight: 20-22g) were purchased from experimental animal center, Air Force Medical University. The mice were randomly divided into different groups: control group: olive oil (MACLIN, China, Cat: 8001-25-0 ), 1ml/kg i.p.; model group [25% CCl4 (MACLIN, China, Cat: 56-23-5) (CCl4 : olive oil = 1:3)，1ml/kg i.p.]; NECA treatment group [ [25% CCl4 , 1ml/kg i.p. + NECA (GLPBIO, Montclair, CA, United States, Cat: GC15304), 0.1mg/kg i.p.]; A1R agonist group [25% CCl4 , 1ml/kg i.p. + CCPA (GLPBIO, Montclair, CA, United States, Cat: GC45773), 0.5mg/kg i.p.]; A2AR agonist group [25% CCl4, 1ml/kg i.p. + CGS21680 (GLPBIO, Montclair, CA, United States, Cat: GC10172), 1mg/kg i.p.]; A2BR agonist group [25% CCl4, 1ml/kg i.p. + BAY-606583 (TargetMol, China, Cat:910487-58-0), 4mg/kg i.p.]; A3R agonist group [25% CCl4 , 1ml/kg i.p. + Namodenoson (TargetMol, China, Cat:163042-96-4), 200ug/kg i.p.]. The CCl4 i.p. was performed twice weekly for 6 weeks. The others i.p. was performed twice weekly from the third week. After the last injection, all mice were fasted overnight. Then, the liver tissues and blood of the mice models were collected.

All experiments were conducted according to animal welfare guidelines by Air Force Medical University’s Institutional Animal Care and Use Committee.

- 1. **Liver function test**

The ALT and AST in serum from mice models were detected by an automatic biochemical analyzer (Beckman Coulter, AU5800, Germany).

- 1. **Pathologic analysis**

Pathological section: Formalin-fixed paraffin-embedded (FFPE) sections of liver tissues were prepared (5μm thick). The H&E staining was performed to evaluate the necrosis, degeneration and inflammatory infiltration. Masson and Sirius red staining were performed to evaluate the content and degree of collagen fiber in hepatic portal area. Pannoramic and CaseViewer 2.4 software (3DHISTECH, Hungary) were used for image acquisition and analysis.

- 1. **Quantitative Real-time PCR Analysis (qRT-PCR)**

Total RNA from cells and liver histiocytic suspension were extracted by using Trizol reagent (TAKARA, Japan, Cat: 9109). The cDNA was generated by using Prime Script™ RT Master Mix (ACCUBATE BIOLOGY, China, AG11728). qRT-PCR reaction system was prepared using BlasTaq^TM^ 2X qPCR Master Mix (abm, Canada, Cat: G891). The reaction was performed on Qiagen Amplifer (Germany, Rotor-gene QMDX 5). All operation was according to the manufacturer’s instructions. The mRNA expression levels in different groups were calculated using GAPDH expression as an internal control. The primers sequences are listed in Table 1.

**1.5 Immunohistochemical (IHC) analysis**

IHC analysis for liver FFPE tissues was carried out to detect the expression of α-SMA and col1α1. In short, anti-col1α1 antibody (1:100 dilution; Servicebio, GB11022-3), and anti- α-SMA antibody (1:1000 dilution; Abcam, ab124964) were used as the primary antibodies. HRP-conjugated anti-rabbit secondary antibody (1:2000 dilution; ZSGB-BIO, Cat: ZB-2301) was used as second antibody. Pannoramic and CaseViewer 2.4 software (3DHISTECH, Hungary) were used for image acquisition and analysis. Image J software was used to calculate the positive areas.

**1.6 Cell Culture**

Human hepatic stellate cell line LX2 was cultured in Dulbecco’s modified Eagle’s medium (DMEM, Gibco, Cat: 12800-017) plus 10% fetal bovine serum (FBS, ExCell Bio, Cat: FSS500) at 37℃ with 5% CO_2_ in a humidified incubator.

**1.7 Cell proliferation assay**

Cell count Kit-8 (CCK8) assay was used to investigate the effects of A1R/A2AR/A2BR/A3R agonist and NECA treatment on LX2 cells proliferation. Cells were uniformly seeded into 96-well plate, with 2000 cells in each well. CCK8 assay was used to investigate the cell viability at different time point. CCK8 reagent was purchased from GLPBIO (GLPBIO, Montclair, CA, USA, Cat: GSK10001). Cells were set into control group and adenosine receptors agonist treatment group (A1R agonist: CCPA; A2AR agonist: CGS21680; A2BR agonist: BAY 60-6583; A3R agonist: Namodenoson; NECA). The drug concentrations were 100μM, 10μM, 10μM, 10nM and 10μM respectively.

**1.8 Statistical Analysis**

All quantitative results have been presented as mean ± standard deviation (SD). All data analyses were performed by using GraphPad prism 8.0. T-test was used to analyze the differences between two independent samples. P<0.05 was considered to be statistically significant.

**2. Results**

**2.1 A1R and A2AR** **agonist aggravated liver fibrosis in CCl4-induced mice**

CCl4-induced mice models were used to investigate the effect of A1R and A2AR agonist. The flow chart of mice experiment was shown in Figure 1A. Compared with control mice, CCl4 i.p. induced the increased levels of serum ALT and AST. Moreover, the serum ALT and AST were more increased in CCl4-induced mice treated by A1R agonist and A2AR agonist (Figure 1B, C).

H&E staining showed that more liver injury and infiltrated inflammatory cells in A1R agonist treated CCl4-mice (Figure 1D). Masson and Sirius-red staining indicated that A1R agonist promote the collagen deposition in liver tissues (Figure 1E, F). Similarly, the pathological section results of liver tissues from mice models also showed the A2R agonist aggravated liver injury, immune cell infiltration and collagen deposition (Figure 1G-I).

**2.2 A2BR and A3R agonist alleviated liver fibrosis in CCl4-induced mice**

We further investigated the effect of A2BR and A3R agonist in liver fibrosis. The procedure of animal experiments was shown in Figure 2A. Compared with CCl4 i.p. mice, A2BR and A3R agonist treatment significantly reduced the ALT and AST levels in CCl4-induced mice (Figure 2B, C). Moreover, the pathological section staining (H&E, Masson, Sirius-red) of liver tissues showed that A2BR agonist reduced the liver injury and collagen deposition (Figure 2D-F). Similarly, A3R agonist i.p. also alleviated liver fibrosis disease activity in CCl4-induced mice models (Figure 2G-I), characterized by the decreasing ALT and AST levels and pathological damage of liver.

**2.3 The effect of A1R/A2AR/A2BR/A3R agonist on HSCs activation and proliferation**

To further explore the potential mechanism of adenosine receptors on liver fibrosis, we investigated the effect of A1R/A2AR/A2BR/A3R agonist i.p. on HSC activation markers in liver tissues. Compared with CCl4-mice, the mRNA expression of α-SMA and Col1α1 were significant increase in A1R/A2AR agonist i.p. mice (Figure 3A, B), while α-SMA and Col1α1 were decreased in A2BR/A3R agonist i.p. mice (Figure 3C, D). In addition, IHC results also showed the promoting effect of A1R/A2AR agonist and inhibiting effect of A2BR/A3R agonist in α-SMA and Col1α-1 protein levels (Figure 3E, F).

Moreover, the A1R and A2AR agonist elevated the expression of SMA and Col1α-1 in LX2, while A2BR and A3R agonist inhibited the expression of α-SMA and Col1α1 in LX2 cells (Figure 3G, H). We further investigated the effects of A1R/A2AR/A2BR/A3R agonist on HSCs proliferation in *vitro*. The results showed that the A1R and A2AR agonist significantly promoted the proliferation of LX2 cells, while the A2BR and A3R agonist inhibited the proliferation of LX2 cells (Figure 3I-L).

**2.4 NECA inhibited HSCs activation and** **alleviate liver fibrosis**

As the different effects of A1R/A2AR/A2BR/A3R agonist i.p. on liver fibrosis, we further investigated the function of NECA (a metabolically stable adenosine analogues) i.p. in CCl4-induced mice (Figure 4A). The results showed that ALT and AST levels were decreased in NECA i.p. mice (vs CCl4-induced mice; Figure 4B, C). H&E staining showed that NECA treatment reduced the liver injury (Figure 4D). Masson and sirius-red staining showed the decreased degree of collagen fiber in the liver from NECA i.p. mice (Figure 4E, F). The expression of SMA and Col1α1 were inhibited by NECA treatment in liver tissues, both in mRNA and protein levels (Figure 4G-J). Moreover, NECA treatment significantly inhibited the expression of SMA and Col1α-1 in LX2 cells (Figure 4K, L). We further investigated the effects of NECA on HSCs proliferation in *vitro*. The results showed that the NECA significantly inhibited the proliferation of LX2 cells (Figure 4M).

**2.5 The different expression levels of A1R/A2AR/A2BR/A3R in HSCs**

As the different effect of A1R/A2AR/A2BR/A3R agonist i.p. on the disease severity, we investigated the expression levels of A1R/A2AR/A2BR/A3R in LX2 cells. The results showed that A2BR was the highest expressed gene among the four adenosine receptors. Notably, the expression levels of A1R/A2AR/A2BR/A3R in LX2 were all decreased by NECA treatment. And the A2BR was still the highest expressed genes after NECA treatment (Figure 4N). These results suggests that the similar effects between NECA and A2BR, which both alleviate liver fibrosis, might be associated with the highest expression levels of A2BR in HSCs.

**Discussion**

Adenosine-receptors pathway play important roles in inflammatory response. Currently, there are four adenosine receptors, which are A1R, A2AR, A2BR and A3R. These four adenosine receptors might play a different role in the progress of different disease, even in the same disease. In this study, we demonstrated that A1R and A2AR activation could aggravate liver fibrosis in CCl4 induced mice models, characterized by increased serum levels of ALT and AST, increased pathological damage and collagen deposition in liver tissues. Consistently, the proliferation and activation of LX2 were significantly promoted by A1R and A2AR agonist treatment. However, the A2BR and A3R agonist treatment significantly alleviated liver fibrosis in mice models, and inhibited the LX2 proliferation and activation. These results revealed the different function of different adenosine receptors during liver fibrosis progress. It is noteworthy that the effect of NECA treatment on liver fibrosis in vivo and LX2 cells in vitro were similar to A2BR agonist.

Although all as the adenosine receptors, accumulated evidence has showed that the different contribution of A1R/A2R/A2BR/A3R during disease development. Firstly, adenosine receptors were involved in multiple disease progress. Studies have demonstrated the function of A1R in brain disease, including epilepsy ^[9, 10]^, alzheimer's disease ^[11]^, parkinson ^[11, 12]^and stroke ^[13]^. The A1R has been a promising therapeutic target for non-opioid analgesic agents to treat neuropathic pain^[14]^. A2AR, A2BR and A3R were mostly involved in cancer development^[15-19]^, inflammation ^[20-22]^, cardiac disease^[23]^ and etc. Secondly, there were different function for these adenosine receptors in the same disease. For example, in hepatic ischemia/reperfusion (IR) injury, A2AR agonist protected the primary steatotic murine hepatocyte from IR damage. By contrast, the A1R agonist enhanced IR damage, intracellular steatosis and oxidative species production^[24]^. Studies have showed the opposite function between A1R and A2AR in central and peripheral nervous systems^[25, 26]^.

In hepatic disease, Zhu et al’s study showed that liver-specific depletion of A1R aggravates while overexpression attenuates diet-induced metabolic-associated steatohepatitis in mice, via regulating SREBPs maturation^[27]^. However, Arroyave-Ospina JC’s study showed that antagonism of A1R could protects against lipo-toxicity in metabolic-dysfunction-associated liver disease, in addition, A1R agonist abolished the protective effect of caffeine^[28]^. For A2AR, both tumor promoting and tumor inhibiting effects in hepatocellular carcinoma (HCC) have been reported. Allard B et al’s study showed that A2AR is a tumor suppressor of non-alcoholic steatohepatitis (NASH)-associated hepatocellular carcinoma. A2AR knockout could promote the development of spontaneous and carcinogen-induced HCC in mice^[29]^. However, Ma XL et al’s study showed that target blockage of A2AR have suppression effects on HCC growth and metastasis^[30]^. In addition, Myojin Y and colleagues found that A2AR inhibition could increase the anti-tumor efficacy of anti-PD1 treatment in HCC mice models, which also indicated the anti-cancer effect of A2AR blockage^[31]^. In NASH, accumulated evidence has showed that A2AR stimulation prevent the development of NASH in mice models, via multilevel inhibition of signals that cause lipo-toxicity and inflammation^[32-34]^. However, in alcoholic liver fibrosis, Chiang DJ’s study showed that A2AR antagonist prevented and reversed liver fibrosis in ethanol-exacerbated liver fibrosis mice models, via suppressing activation of HSC^[35]^. In addition to A2AR, studies have showed that A2BR or A3R stimulation could ameliorate the fibrotic progress in NASH mice models ^[36, 37]^. Taken together, above studies demonstrated that the roles of adenosine receptors were complex and multifaceted in liver disease models with different backgrounds.

**Conclusion**

In conclusion, our study demonstrated the different effects among A1R, A2AR, A2BR and A3R in liver fibrosis, might via regulating HSC activation, at least in part. As the complex function of adenosine receptors in liver fibrosis, the safety and potential side effect of adenosine target therapy needed more attention in clinical trial study.

**Figure legends**

**Figure 1. A1R and A2AR agonist aggravated liver fibrosis in CCl4-inducedmice.** A: low chart of animal experiments. (B, C) Serum ALT and AST levels in control, CCl4-mice, CCPA and CGS21680 treated mice. (D-I): Representative liver tissue sections of control，CCl4，CCPA and CGS21680 treatment group were detected by HE、Masson and Sirius Red staining, scale bar, 50μm; magnification, 20x. The positive areas statistic of Masson and Sirius Red staining were measured by Image J software. *P < 0.05, **P < 0.01, ***P < 0.0005, ****P < 0.0001.

**Figure 2. A2BR and A3R agonist alleviated liver fibrosis in CCl4-induced mice.** A: low chart of animal experiments. (B, C) Serum ALT and AST levels in control, CCl4-mice, BAY 60-6583 and Namodenoson treated mice. (D-I) Representative liver tissue sections of control、CCl4 ,BAY 60-6583 and Namodenoson treatment group were detected by HE、Masson and Sirius Red staining, scale bar, 50μm; magnification, 20x. The positive areas statistic of Masson and Sirius Red staining were measured by Image J software. **P* < 0.05, ***P* < 0.01, ****P* < 0.0005, *****P* < 0.0001.

**Figure 3. The effect of A1R/A2AR/A2BR/A3R agonist on HSCs activation and proliferation. (**A-F)**:** The expression levels of α-SMA and col1α1 in liver tissues from olive、CCl4 , CCPA, CGS21680, BAY60-6583 and Namodenoson treatment mice were analyzed by qRT-PCR and immunohistochemical (IHC) staining analysis. scale bar, 50μm; magnification, 20x. (G, H): The expression levels of α-SMA and col1α1 in LX2 were analyzed by qRT-PCR. (I-L): CCK8 showed the effect of CCPA, CGS21680, BAY60-6583 and Namodenoson treatment on LX2 proliferation. **P* < 0.05, ***P* < 0.01, ****P* < 0.0005, *****P* < 0.0001.

**Figure 4. NECA inhibited HSCs activation and alleviate liver fibrosis.** A: low chart of animal experiments. (B-C): Serum ALT and AST levels in control, CCl4-mice and NECA treated mice. (D-F): Representative liver tissue section of control、CCl4 and NECA treatment group were detected by HE、Masson and Sirius Red staining, scale bar, 50μm; magnification, 20x. (G-J): The expression levels of α-SMA and col1α1 in olive、CCl4 NECA treatment mice were analyzed by qRT-PCR and immunohistochemical (IHC) staining analysis. scale bar, 50μm; magnification, 20x. The positive areas statistic of Masson and Sirius Red staining were measured by Image J software. (K-L): The expression levels of α-SMA and col1α1 in LX2 were analyzed by qRT-PCR. M: CCK8 showed the effect of NECA treatment on LX2 proliferation. N: The different expressionof A1R, A2AR, A2BR and A3R with NECA treatment in LX2 cells were analyzed by qRT-PCR. **P* < 0.05, ***P* < 0.01, ****P* < 0.0005, *****P* < 0.0001.

**Ethics statement**

The animal study was approved by Air Force Medical University’s Institutional Animal Care and Use Committee. The study was conducted in accordance with the local legislation and institutional requirements.

**Author contributions**

LY: Data curation, Formal analysis,Writing-original draft; ZG：Formal analysis, Methodology, Writing – review & editing; XW: Software, Data curation, Writing-original draft; XW: Data curation, Writing-original draft; SL: Data curation, Writing-original draft; KD: Supervision, Writing – review & editing; XZ: Methodology, Project administration, Writing – review & editing.

**Acknowledgments**

We would like to acknowledge the reviewers for their helpful comments on this paper.

**Conflict of interest**

The authors declare that the research was conducted in the absence of any commercial or financial relationships that could be construed as a potential conflict of interest.

The author(s) declared that they were an editorial board member of Frontier, at the time of submission. This had no impact on the peer review process and the final decision.

**References**

1. Kisseleva, T. and D. Brenner, *Molecular and cellular mechanisms of liver fibrosis and its regression.* Nat Rev Gastroenterol Hepatol, 2021. **18**(3): p. 151-166.

2. Chanda, D., et al., *Developmental pathways in the pathogenesis of lung fibrosis.* Mol Aspects Med, 2019. **65**: p. 56-69.

3. Parola, M. and M. Pinzani, *Liver fibrosis: Pathophysiology, pathogenetic targets and clinical issues.* Mol Aspects Med, 2019. **65**: p. 37-55.

4. Pei, Q., Q. Yi, and L. Tang, *Liver Fibrosis Resolution: From Molecular Mechanisms to Therapeutic Opportunities.* Int J Mol Sci, 2023. **24**(11).

5. Caligiuri, A., et al., *Cellular and Molecular Mechanisms Underlying Liver Fibrosis Regression.* Cells, 2021. **10**(10).

6. Li, X., et al., *Adenosine at the Interphase of Hypoxia and Inflammation in Lung Injury.* Front Immunol, 2020. **11**: p. 604944.

7. Peleli, M., et al., *Pharmacological targeting of adenosine receptor signaling.* Mol Aspects Med, 2017. **55**: p. 4-8.

8. Fausther, M., *Extracellular adenosine: a critical signal in liver fibrosis.* Am J Physiol Gastrointest Liver Physiol, 2018. **315**(1): p. G12-g19.

9. Masino, S.A., et al., *A ketogenic diet suppresses seizures in mice through adenosine A₁ receptors.* J Clin Invest, 2011. **121**(7): p. 2679-83.

10. Amorim, B.O., et al., *Effects of A1 receptor agonist/antagonist on spontaneous seizures in pilocarpine-induced epileptic rats.* Epilepsy Behav, 2016. **61**: p. 168-173.

11. Rivera-Oliver, M. and M. Díaz-Ríos, *Using caffeine and other adenosine receptor antagonists and agonists as therapeutic tools against neurodegenerative diseases: a review.* Life Sci, 2014. **101**(1-2): p. 1-9.

12. Jakova, E., et al., *Adenosine A1 receptor ligands bind to α-synuclein: implications for α-synuclein misfolding and α-synucleinopathy in Parkinson's disease.* Transl Neurodegener, 2022. **11**(1): p. 9.

13. Liston, T.E., et al., *Adenosine A1R/A3R (Adenosine A1 and A3 Receptor) Agonist AST-004 Reduces Brain Infarction in a Nonhuman Primate Model of Stroke.* Stroke, 2022. **53**(1): p. 238-248.

14. Draper-Joyce, C.J., et al., *Positive allosteric mechanisms of adenosine A(1) receptor-mediated analgesia.* Nature, 2021. **597**(7877): p. 571-576.

15. Shi, L., et al., *Adenosine interaction with adenosine receptor A2a promotes gastric cancer metastasis by enhancing PI3K-AKT-mTOR signaling.* Mol Biol Cell, 2019. **30**(19): p. 2527-2534.

16. Willingham, S.B., et al., *A2AR Antagonism with CPI-444 Induces Antitumor Responses and Augments Efficacy to Anti-PD-(L)1 and Anti-CTLA-4 in Preclinical Models.* Cancer Immunol Res, 2018. **6**(10): p. 1136-1149.

17. Cekic, C., et al., *Adenosine A2B receptor blockade slows growth of bladder and breast tumors.* J Immunol, 2012. **188**(1): p. 198-205.

18. Vecchio, E.A., et al., *Ligand-Independent Adenosine A2B Receptor Constitutive Activity as a Promoter of Prostate Cancer Cell Proliferation.* J Pharmacol Exp Ther, 2016. **357**(1): p. 36-44.

19. Harvey, J.B., et al., *CD73's Potential as an Immunotherapy Target in Gastrointestinal Cancers.* Front Immunol, 2020. **11**: p. 508.

20. Mediero, A., et al., *Brief Report: Methotrexate Prevents Wear Particle-Induced Inflammatory Osteolysis in Mice Via Activation of Adenosine A2A Receptor.* Arthritis Rheumatol, 2015. **67**(3): p. 849-55.

21. Barletta, K.E., et al., *Adenosine A(2B) receptor deficiency promotes host defenses against gram-negative bacterial pneumonia.* Am J Respir Crit Care Med, 2012. **186**(10): p. 1044-50.

22. D'Antongiovanni, V., et al., *Glial A(2B) Adenosine Receptors Modulate Abnormal Tachykininergic Responses and Prevent Enteric Inflammation Associated with High Fat Diet-Induced Obesity.* Cells, 2020. **9**(5).

23. Lu, Z., et al., *Adenosine A3 receptor deficiency exerts unanticipated protective effects on the pressure-overloaded left ventricle.* Circulation, 2008. **118**(17): p. 1713-21.

24. Alchera, E., et al., *Ischemia/Reperfusion Injury of Fatty Liver Is Protected by A2AR and Exacerbated by A1R Stimulation through Opposite Effects on ASK1 Activation.* Cells, 2021. **10**(11).

25. Deb, P.K., et al., *Medicinal Chemistry and Therapeutic Potential of Agonists, Antagonists and Allosteric Modulators of A1 Adenosine Receptor: Current Status and Perspectives.* Curr Pharm Des, 2019. **25**(25): p. 2697-2715.

26. Melani, A., et al., *The selective A2A receptor antagonist SCH 58261 protects from neurological deficit, brain damage and activation of p38 MAPK in rat focal cerebral ischemia.* Brain Res, 2006. **1073-1074**: p. 470-80.

27. Zhu, W., et al., *Activation of hepatic adenosine A1 receptor ameliorates MASH via inhibiting SREBPs maturation.* Cell Rep Med, 2024. **5**(3): p. 101477.

28. Arroyave-Ospina, J.C., et al., *Protective effects of caffeine against palmitate-induced lipid toxicity in primary rat hepatocytes is associated with modulation of adenosine receptor A1 signaling.* Biomed Pharmacother, 2023. **165**: p. 114884.

29. Allard, B., et al., *Adenosine A2A receptor is a tumor suppressor of NASH-associated hepatocellular carcinoma.* Cell Rep Med, 2023. **4**(9): p. 101188.

30. Ma, X.L., et al., *CD73 promotes hepatocellular carcinoma progression and metastasis via activating PI3K/AKT signaling by inducing Rap1-mediated membrane localization of P110β and predicts poor prognosis.* J Hematol Oncol, 2019. **12**(1): p. 37.

31. Myojin, Y., et al., *Adenosine A2a receptor inhibition increases the anti-tumor efficacy of anti-PD1 treatment in murine hepatobiliary cancers.* JHEP Rep, 2024. **6**(1): p. 100959.

32. Zhou, J., et al., *Mice lacking adenosine 2A receptor reveal increased severity of MCD-induced NASH.* J Endocrinol, 2019.

33. Alchera, E., et al., *Adenosine A2a receptor stimulation blocks development of nonalcoholic steatohepatitis in mice by multilevel inhibition of signals that cause immunolipotoxicity.* Transl Res, 2017. **182**: p. 75-87.

34. Imarisio, C., et al., *Adenosine A(2a) receptor stimulation prevents hepatocyte lipotoxicity and non-alcoholic steatohepatitis (NASH) in rats.* Clin Sci (Lond), 2012. **123**(5): p. 323-32.

35. Chiang, D.J., et al., *Adenosine 2A receptor antagonist prevented and reversed liver fibrosis in a mouse model of ethanol-exacerbated liver fibrosis.* PLoS One, 2013. **8**(7): p. e69114.

36. Li, H., et al., *Modafinil exerts anti-inflammatory and anti-fibrotic effects by upregulating adenosine A(2A) and A(2B) receptors.* Purinergic Signal, 2023.

37. Fishman, P., et al., *The A3 adenosine receptor agonist, namodenoson, ameliorates non‑alcoholic steatohepatitis in mice.* Int J Mol Med, 2019. **44**(6): p. 2256-2264.
