# Supplementary material for: The different effects of four adenosine receptors in liver fibrosis
Source: Front Pharmacol. 2024 Sep 3;15:1424624. doi: 10.3389/fphar.2024.1424624 (PMC11405188; doi:10.3389/fphar.2024.1424624)
Supplement: Supplementary file 5 [file DataSheet2.ZIP › files-2/DK 受体与肝病.doc]

**A1R**

4.抑制CD73/A1R表达，通过下调PLC-IP3-Ca2+/DAG-PKC表达，减少α-SMA和col1a1表达，促进HSC-T6凋亡，缓解ALF。 PMID 35784730

5.肝脏A1R激活通过抑制胆固醇调节原件结合蛋白SREBP成熟抑制脂肪生成和成熟，缓解饮食相关脂肪肝。PMID:38508143

33. 在代谢功能障碍相关肝病中，抑制A1R可以促进肝脏脂滴形成，降低线粒体DNA ROS产生，降低肝脏脂毒性。PMID:37423170

**A2AR**

8. A2AR抑制非酒精性脂肪肝相关肝癌的发生，A2AR^-/-^小鼠发生肥胖，NASH，炎症，导致原发性肝癌；肝癌患者中，低表达A2AR与肝硬化、肝炎及不良预后相关。PMID 37729873

9. A2AR促进经血来源的间充质干细胞抑制TLR4介导的PI3K/Akt/mTOR/IKK信号通路，减轻急性肝衰导致的肝损伤，PMID:33729623

11.在肥胖相关NASH中，阻断A2AR增加巨噬细胞炎症答复，促进MCD介导NASH。PMID:31505462

16. CD73/A2AR通过激活PI3K/AKT信号通路促进HCC的进展和转移，抗A2AR抗体可以抑制HCC进展和转移。 PMID:30971294

31. A2AR抑制剂联合抗PD1治疗通过引起T细胞活化缓解肝癌。PMID:38192537

34. A2AR通过恢复血管内皮的抗炎、屏障和血管舒张反应以及抑制白细胞与内皮的黏附，缓解肝硬化相关肝肾内皮功能障碍、门静脉高压、肾脏低灌注和肾功能不全。PMID:37059491

52. A2AR抑制内脏脂肪组织来源的致病信号，从而改善高脂饮食(HFD)诱导的NASH小鼠的肝功能和心功能障碍。PMID:38252682

53. A2AR通过减少Th17细胞扩增和抑制IL-17诱导的JNK依赖性脂毒性加重,抑制NASH发展。PMID:28011152

54. A2AR激动剂通过抑制JNK-1/2, 可降低脂毒性并改善NASH PMID:22439844

55. A2AR激动剂促进HSC活化，而A2AR拮抗剂可以预防和逆转乙醇诱导肝纤维化PMID:23874883

56. 咖啡因通过A2AR调控cAMP/PKA/SRC/ERK1/2/P38 MAPK信号通路，抑制乙醛诱导的HSC活化PMID:24682220

**A2BR**

17. (临床研究)A2BR在HCC中表达上调，A2BR的表达水平与HCC的肿瘤进展相关。 PMID:16844405

**A3R**

21. 肝癌肿瘤组织和PBMC中A3R表达增加，A3R激动剂CF102通过下调Wnt和NF-κB通路介导肝癌细胞凋亡。PMID:18636149
