# Supplementary material for: The different effects of four adenosine receptors in liver fibrosis
Source: Front Pharmacol. 2024 Sep 3;15:1424624. doi: 10.3389/fphar.2024.1424624 (PMC11405188; doi:10.3389/fphar.2024.1424624)
Supplement: Supplementary file 5 [file DataSheet2.ZIP › files-2/WX 不同受体-相同疾病-不同作用.doc]

1.A2AR激动剂CGS21680通过激活ASK1/JNK通路减轻脂肪肝缺血再灌注损伤，胞内脂质代谢及氧化应激，A1R激动剂CCPA加重。PMID:34831394

2.酒精性肝纤维化小鼠中，4种受体表达增加，抑制四种受体是一种ALF的潜在治疗方法。 PMID:37385371

3. adenosine-conjugated nanoparticles促进cAMP产生，缓解大鼠骨关节炎，cAMP产生可被A2AR/A2BR拮抗剂阻断。 PMID:31092864

38. cAMP/PKA/CREB信号通过A2AR和A1R可能介导乙醛诱导的HSC的活化。 PMID:25956975

19. 缺氧状态下，A2BR在结直肠癌中过表达，促进结肠癌细胞生长。PMID:20619442

23. 缺氧状态下，A2BR在结直肠癌中表达上调，促进癌细胞增殖，这一作用可被A2BR拮抗抑制PMID:20619442

24. 在CAF(肿瘤相关纤维母细胞)富集的结肠癌肿瘤中，同时抑制A2AR、A2BR信号通路可以增强抗肿瘤免疫。PMID:31980601

40. （PMID:35335254）

在中枢神经系统损伤（脑缺血），A1Rs通过抑制突触前电压门控钙通道(VGCCs)来抑制神经递质释放1-PMID:8096697，维持细胞处于低能量消耗状态和抑制兴奋性毒性损伤的谷氨酸过度释放介导神经保护作用2-PMID:6149943；

A2AR促进钙离子摄取，促进神经递质释放3-PMID:8884224。 A2AR抑制电压依赖性钾通道，促进细胞兴奋性和神经递质释放，加重缺血/缺氧条件下的兴奋性毒性损伤4-PMID:12044450

A2BR促进谷氨酸释放，增强神经毒性，5-PMID：29740323，抑制少突胶质细胞K+电流和细胞分化，调节1-磷酸鞘氨醇信号通路，引起神经元损伤6-PMID:32251679

缺氧时，A3R - / -小鼠神经变性增加7-PMID:12825837，大鼠皮质神经元中激活A3Rs抑制兴奋性神经递质释放，促进神经保护8-PMID:12808203；

脑缺血10或20分钟前慢性给予A3受体激动剂（IB-MECA）治疗，可改善缺血后的脑血液循环，生存率和神经元保护，而急性给予IB-MECA时，观察到相反的效应，即缺血后血流受损、死亡率增加和海马内广泛的神经元破坏9-PMID:7821362 ； 通过氧糖剥夺 (OGD)建立体外脑缺血模型表明，在CA1海马区，阻断A3Rs可防止由氧糖剥夺引起的突触衰竭和兴奋性毒性损伤，增强对缺血损伤的抵抗力10-PMID:16415905

41.（PMID:35335254）长时间OGD期间，A3R刺激在损伤的第一阶段具有保护作用，与A1R一起参与(Gi介导)兴奋性神经传递的抑制PMID:17626785，而在后期，长时间的受体激活可能导致(Gq介导)细胞内钙积累，导致细胞损伤PMID:26581499

42. （PMID:35335254）在对小鼠进行持续性疼痛刺激时，A1R激动剂介导局部外周镇痛效应，A2AR激动剂促进疼痛反应。1-PMID:1438021；

在脊髓神经根性撕脱痛(spinal neuropathic avulsion pain, SNAP)大鼠模型中，单次鞘内注射A2AR激动剂可以逆转脊髓损伤引起的疼痛，2-PMID:29366930；与这一发现不同的是，A2AR拮抗剂可以减轻奥沙利铂引起的神经毒性，减少小胶质细胞中ROS产生，缓解神经痛。3- PMID:31453698

骨髓细胞中激活A2BR通过可溶性IL-6受体信号传导，导致疼痛感受器过度兴奋，促进慢性神经痛4-PMID:27320922；在局部炎症和全身炎症两种动物模型中，A2BR拮抗剂显著降低炎性细胞因子IL-6、TNF-α和ROS水平，并通过减少白细胞浸润减轻腹膜炎症 5-PMID:33385856

A3R激动剂（IB-MECA）通过抑制脊髓NADPH氧化酶和两个下游氧化还原依赖系统的激活来减轻紫杉醇诱导的神经病理性疼痛。第一种依赖于抑制氧化还原敏感的转录因子(NFκB)和丝裂原活化蛋白激酶(ERK和p38)，从而减少神经兴奋性/促炎细胞因子(TNF-α， IL-1β)的产生，促进神经保护/抗炎IL-10的形成。第二种涉及抑制氧化还原介导的翻译后酪氨酸硝化和胶质细胞限制性蛋白的修饰(失活)，这些蛋白在调节突触谷氨酸稳态中起关键作用:谷氨酸转运体GLT-1和谷氨酰胺合成酶6-PMID:25242567

A3R激动剂通过激活CD4+ T细胞上的A3R，释放IL-10， 降低背部神经节（DRG）神经元的兴奋性，缓解神经痛7-PMID:33621215

49. A1R激动剂可降低睫状无色素上皮细胞的外流阻力，从而降低眼压; A1R, A2AR激动剂可降低血管阻力，增加视网膜和视盘血流量。A1R激动剂和A2AR拮抗剂可促进缺血后视网膜功能的恢复。A3R可减轻伴随P2X(7)受体激活的钙升高和视网膜神经节细胞死亡。3-PMID:23328492
