# Supplementary material for: The different effects of four adenosine receptors in liver fibrosis
Source: Front Pharmacol. 2024 Sep 3;15:1424624. doi: 10.3389/fphar.2024.1424624 (PMC11405188; doi:10.3389/fphar.2024.1424624)
Supplement: Supplementary file 5 [file DataSheet2.ZIP › files-2/WXN LSM 相同受体-不同疾病-不同作用.doc]

A1R

43. PMID:31333094

心血管：

在大鼠心肌细胞H9c2中，A1R激动剂通过多蛋白激酶依赖途径刺激组织型转谷氨酰胺酶(TG2)活性，抑制缺氧诱导的LDH释放，保护心肌细胞 1-PMID:27005940；

A1R增加了房室结细胞速率依赖性激活失败率，引起房室结细胞的超极化，抑制房室结细胞的兴奋性，引起二度房室传导阻滞 2- PMID:8635227

44. PMID:31333094

肾病：

A1R拮抗剂在实验动物和人类中诱导利尿和利钠作用，这一作用主要是由于近端肾小管负责过滤后Na^+^和液体的重吸收，抑制近端肾小管A1R可减少液体重吸收，降低盐敏感性高血压模型中升高的血压。1-PMID:25345761

A1R激动剂通过减少肾小管坏死、凋亡和炎症，在肾缺血再灌注损伤中发挥保护作用，而A1R拮抗剂预处理的C57BL/6小鼠的肾功能明显恶化，炎症标志物、坏死和凋亡的表达增加 2-PMID:14694162；3-PMID:14600029

内源性A1AR激活通过调节HSP27信号通路在肾近端小管中产生细胞保护作用；4-PMID:17429344

A1R激动剂CCPA通过细胞外信号调节激酶途径诱导IL-11 合成，减轻缺血性急性肾损伤5-PMID:23813214

A1R激活通过诱导HSP27产生，减少caspase-3激活和DNA断裂，抑制细胞凋亡，保护肾脏免受缺血/再灌注损伤 6-PMID:29480441

45. PMID:31333094

抗痉挛：

A1R激动剂降低了慢性癫痫大鼠的海马兴奋性，但没有降低自发性反复癫痫发作的频率，而A1R拮抗剂增加了两者 1-PMID:27371881

腺苷通过激活突触前A1R, 降低电压依赖性Ca2+电流、激活K+电流或通过Ca2+内流下游机制来抑制递质释放, 使神经元的超极化性兴奋性传递减少。2- PMID:8185949

腺苷激活突触前A1受体，调节P/Q-和n -型突触前电压依赖性Ca2+通道，控制苔藓纤维突触的递质释放。3- PMID:17478533

重复刺激A1R产生的抗惊厥效应具有耐受性，A2AR激活可以维持抗惊厥效应。4-PMID:8750698

46. PMID:31333094

神经：

A1R可以抑制左旋多巴诱导的运动障碍1-PMID:25173217，破坏A1R可能导致神经功能障碍2-PMID:27134041

在亨廷顿舞蹈症模型小鼠R6/2的皮质纹状体脑片中，A1R激动剂通过减少突触传递，抑制谷氨酸释放，保护神经3-PMID:25132555，A1R拮抗剂增强了神经元损伤， 而A2AR拮抗剂减轻了神经元损伤。4-PMID:14645494。

在3-硝基丙酸(3NP)诱导的亨廷顿舞蹈病大鼠模型中，A1R激动剂可以减轻肢体肌张力障碍和纹状体变性5-PMID:12388620。

A1R激动剂抑制肿瘤生长。然而，当从切片中去除小胶质细胞时，激动剂促进了肿瘤生长。因此，腺苷通过A1R作用于小胶质细胞抑制胶质母细胞瘤的生长。6-PMID:16951167

使用腺苷受体拮抗剂(如咖啡因)阻断A1R和A2AR,可减轻脊髓损伤(SCI)或卒中(脑梗死)以及其他神经退行性疾病(如帕金森病和阿尔茨海默病)引起的物理、细胞和分子损伤7-PMID:24530739

缺血性脑卒中 PMID: 34802248

47. PMID:31333094

哮喘：

腺苷可通过激活A1R介导支气管收缩、促进促炎功能、增加内皮细胞的通透性以及控制黏蛋白的产生，引起哮喘1-PMID:15345696。。

A1R拮抗剂PBF-680治疗轻中度哮喘的疗效正在进行临床评估(NCT02635945)。

48. PMID:31333094

胃肠疾病：

A1R拮抗剂可增强下消化道推进力，促进排便，但不引起腹泻。1-PMID:7494374

A1R通过降低LPS诱导TNF-α，增加IL-10的浓度，在某些肠道炎症性疾病(如溃疡性结肠炎和克罗恩病)中发挥抗炎作用2-PMID:8906843

A1R激动剂可减轻多发性硬化模型的神经炎症和脱髓鞘，A1 R^-/-^小鼠脱髓鞘加重，轴索损伤，小胶质细胞/巨噬细胞活化增强，神经炎症加重3-PMID:14960625；

A1R激动剂CCPA通过减少炎症、坏死和凋亡，改善缺血再灌注损伤后的肾功能4-PMID：14694162

在正常结肠中，A1R和A2AR在神经元水平上参与运动功能的抑制控制。在肠道炎症状态下，A1R失去其调节作用，而A2AR可增强对结肠神经运动的抑制控制。5-PMID:21034735

49. PMID:31333094

眼病：

在任何物种中A3R增加眼压，并在小鼠和大型哺乳动物中,A1R降低眼压和A2AR增加眼压。1-PMID:11564641

A1R激动剂通过激活G(i/o) > PLC > PKCalpha > c-Raf > mitogen-activated protein kinase kinase > ERK1/2，导致MMP-2的分泌，从而促进小梁网细胞（trabecular meshwork cells）房水流出。2-PMID:17015637

A1R激动剂可降低睫状无色素上皮细胞的外流阻力，从而降低眼压; A1R, A2AR激动剂可降低血管阻力，增加视网膜和视盘血流量。A1R激动剂和A2AR拮抗剂可促进缺血后视网膜功能的恢复。A3R可减轻伴随P2X(7)受体激活的钙升高和视网膜神经节细胞死亡。3-PMID:23328492

50. PMID:31333094

II型糖尿病：

抑制A1R通过调节氧化应激和炎症反应，改善代谢、胰岛素分泌和血管功能，预防糖尿病发生或进展1-PMID:25835725

A1R或A2BR抑制剂保护小鼠免于脂肪肝，在酒精性脂肪肝中， A1R促进脂肪酸合成和A2BR减少脂肪酸代谢2-PMID:19221436。

在瘦和肥胖动物中，较低胰岛素水平下，A1AR拮抗剂可以促进葡萄糖耐量的增加。3-PMID:9486158

52. PMID:31333094

肿瘤：

A1R拮抗剂DPCPX通过调节ERK/JNK通路抑制肾癌细胞增殖和迁移，诱导细胞凋亡和s期细胞周期阻滞，进而抑制肿瘤进展 1-PMID:28950257

A1R拮抗剂DPCPX可以通过抑制雌激素受体-α及其内源性配体雌二醇(E2)依赖的转录活性抑制乳腺癌细胞MCF-7增殖。利用siRNA敲除A1R可以降低MCF7细胞的增殖能力，而A1R过表达可以增强细胞的增殖能力2-PMID:19935720。

在人乳腺癌细胞MDA-MB-468中，利用siRNA敲除A1R的表达可以抑制细胞增殖，促进细胞凋亡3-PMID:16294023相反，Hosseinzadeh等人的研究显示A1R激动剂(CHA或R-PIA)在人肝癌细胞HepG2和结直肠癌细胞CACO2中具有抑制增殖作用 4-PMID:19079533；A1R通过激活caspase-3、-8和-9诱导人结肠癌CW2细胞凋亡，从而抑制人结肠癌细胞CW2的生长。5- PMID:19822392；

A2AR

10. （临床研究）与正常对照相比，CD73在胰管腺癌pancreatic ductal adenocarcinoma (PDAC) 和壶腹癌ampullary carcinoma (AC)中过表达，A2AR仅在PDAC中高表达。在PDAC中，CD73过表达与较长的总生存期显著相关（p=0.018），A2AR高表达与高分级(p= 0.001)及晚期(p= 0.01)相关。PMID:35485681

12. （综述）肥大细胞A3R活化促进肿瘤微环境重塑和血管生成，增加M2型巨噬细胞的数量，促进肿瘤中髓源性抑制细胞(MDSCs)的聚集；Treg A2AR激活可增强其免疫抑制活性(例如抑制效应T细胞)。NK细胞、树突状细胞和效应T细胞上的A2AR和/或A2BR活化会抑制其抗肿瘤活性。PMID:32351498

A3R agonist, CF102, is being evaluated as second-line therapy for HCC (NCT02128958)

13. 与野生型小鼠相比，A2AR^-/-^小鼠淋巴瘤肿瘤疫苗的反应增强;，这些小鼠的肿瘤特异性CD8+ T细胞增加，存活率提高PMID:22116345

14. 基因或药物靶向抑制A2AR均显著提高了CAR T细胞的疗效，尤其是与PD-1阻断联合应用时, 这与CD8+ CAR T细胞产生的细胞因子增加以及CD8+和CD4+ CAR T细胞的活化增加相关。 PMID:28165340

15. A2AR在人胃癌组织中表达增加，并与肿瘤的低分化、晚期、淋巴结阳性和较差的患者预后相关。腺苷通过与A2AR相互作用来增强PI3K-AKT-mTOR通路，从而促进胃癌细胞的侵袭和转移。 PMID:31339445

22. 在结肠癌细胞MC38中， A2AR抑制剂CPI-444通过刺激T细胞信号，IL2和IFNγ产生介导抗肿瘤免疫答复，CPI-444联合抗PD1，抗CTLA-4增强肿瘤抑制效果。 PMID：30131376

30 咖啡因通过抑制A2AR/cAMP/PKA通路和下调sirtuin1来增强组蛋白乙酰化和胆固醇合成的基因的表达，促进雄性子代大鼠肝脏胆固醇合成和高胆固醇血症。PMID:30825513

32. 创伤失血性休克中，A2AR减少肺中性粒细胞、肺促炎细胞因子（IL-6、TNFα、）及肺泡灌洗液的产生。抑制剂或者A2AR^-/-^可以逆转，并且抗凋亡marker显著降低。PMID:36018304

35. 死亡供者肾组织中，TNF-α/NF-κB通路促进A2AR表达增加，进而激活PKA-CREB轴，诱导M2巨噬细胞，产生TGF-β1，最终导致纤维化。PMID:33233484

39.甲氨蝶呤通过刺激A2A受体阻止了微粒植入部位的炎症浸润，增加了成骨细胞的数量，减少炎症部位的破骨细胞的数量，间接减少炎性骨溶解。PMID:25533750

A2BR

18. 阻断A2BR通过增强DC的活化和CXCR3依赖的抗肿瘤反应,抑制膀胱癌细胞MB49和乳腺癌细胞4T1的生长和转移。PMID:22116822

20. A2BR促进前列腺癌细胞的增殖。PMID:26791603

36. 创伤后失血性休克中，A2BR敲除加重肺损伤、促进炎症细胞因子和趋化因子产生以及巨噬细胞和中性粒细胞在肺内的浸润和聚集。PMID:37438813

37. A2BR激动剂BAY60-6583可降低TLR4表达、IL-1β、胶质细胞源性神经营养因子（GDNF）和神经肽P物质（SP）释放，调节异常速激肽反应并预防与高脂饮食诱导的肥胖相关的肠道炎症。PMID:32443525

A3R

40. 缺氧时，A3R - / -小鼠神经变性增加7-PMID:12825837，大鼠皮质神经元中激活A3Rs抑制兴奋性神经递质释放，促进神经保护8-PMID:12808203；

脑缺血10或20分钟前慢性给予A3受体激动剂（IB-MECA）治疗，可改善缺血后的脑血液循环，生存率和神经元保护，而急性给予IB-MECA时，观察到相反的效应，即缺血后血流受损、死亡率增加和海马内广泛的神经元破坏9-PMID:7821362 ； 通过氧糖剥夺 (OGD)建立体外脑缺血模型表明，在CA1海马区，阻断A3Rs可防止由氧糖剥夺引起的突触衰竭和兴奋性毒性损伤，增强对缺血损伤的抵抗力10-PMID:16415905

41. （PMID:35335254）长时间OGD期间，A3R刺激在损伤的第一阶段具有保护作用，与A1R一起参与(Gi介导)兴奋性神经传递的抑制PMID:17626785，而在后期，长时间的受体激活可能导致(Gq介导)细胞内钙积累，导致细胞损伤PMID:26581499
42. A3R激动剂（IB-MECA）通过抑制脊髓NADPH氧化酶和两个下游氧化还原依赖系统的激活来减轻紫杉醇诱导的神经病理性疼痛。第一种依赖于抑制氧化还原敏感的转录因子(NFκB)和丝裂原活化蛋白激酶(ERK和p38)，从而减少神经兴奋性/促炎细胞因子(TNF-α， IL-1β)的产生，促进神经保护/抗炎IL-10的形成。第二种涉及抑制氧化还原介导的翻译后酪氨酸硝化和胶质细胞限制性蛋白的修饰(失活)，这些蛋白在调节突触谷氨酸稳态中起关键作用:谷氨酸转运体GLT-1和谷氨酰胺合成酶6-PMID:25242567

A3R激动剂通过激活CD4+ T细胞上的A3R，释放IL-10， 降低背部神经节（DRG）神经元的兴奋性，缓解神经痛7-PMID:33621215

49. A3R可减轻伴随P2X(7)受体激活的钙升高和视网膜神经节细胞死亡。3-PMID:23328492
