# Supplementary material for: The different effects of four adenosine receptors in liver fibrosis
Source: Front Pharmacol. 2024 Sep 3;15:1424624. doi: 10.3389/fphar.2024.1424624 (PMC11405188; doi:10.3389/fphar.2024.1424624)
Supplement: Supplementary file 5 [file DataSheet2.ZIP › files-2/ZXM 同一受体-同一疾病.doc]

6.在中枢神经系统疾病中，白果内酯通过A1R介导的肌动蛋白结合蛋白磷酸化来改变紧密连接的超微结构，可逆性促进血脑屏障的通透性。PMID 32312522

7.银杏叶通过A1R信号通路诱导ERM/MLC磷酸化，增加细胞间连接间隙，可逆性增加血脑屏障通透性。PMID:31541722

19. 缺氧状态下，A2BR在结直肠癌中过表达，促进结肠癌细胞生长。PMID:20619442

23. 缺氧状态下，A2BR在结直肠癌中表达上调，促进癌细胞增殖，这一作用可被A2BR拮抗抑制PMID:20619442

25.（临床研究） A3R在人结直肠癌组织中过表达PMID:15355922

26. 结直肠癌中，A1R,A2AR,A2BR表达较低，A3R表达高，并通过细胞外信号调节蛋白激酶1/2 (ERK1/2)刺激肿瘤生长。PMID:17348028

27. A3R通过促进IL-12产生和增强NK细胞活性，抑制结肠癌细胞增殖。PMID:14562031

28-29. A3R通过下调PKB/Akt, NF-κB, cyclin D1, 上调caspase-3抑制结肠癌发展。PMID:14691449，PMID:15720820
